# SHP2‐Triggered Endothelial Cell Activation Fuels Estradiol‐Independent Endometrial Sterile Inflammation

**DOI:** 10.1002/advs.202403038

**Published:** 2024-09-05

**Authors:** Jie Pan, Jiao Qu, Wen Fang, Lixin Zhao, Wei Zheng, Linhui Zhai, Minjia Tan, Qiang Xu, Qianming Du, Wen Lv, Yang Sun

**Affiliations:** ^1^ State Key Laboratory of Pharmaceutical Biotechnology and Nanjing Drum Tower Hospital the Affiliated Hospital of Nanjing University Medical School School of Life Sciences Nanjing University 163 Xianlin Avenue Nanjing 210023 China; ^2^ General Clinical Research Center Nanjing First Hospital Nanjing Medical University Nanjing 210006 China; ^3^ School of Basic Medicine & Clinical Pharmacy China Pharmaceutical University Nanjing 210009 China; ^4^ Department of Gynecology Tongde Hospital of Zhejiang Province 234 Gucui Road Hangzhou Zhejiang 310012 China; ^5^ Jiangsu Key Laboratory of New Drug Research and Clinical Pharmacy Xuzhou Medical University 209 Tongshan Road Xuzhou Jiangsu 221004 China; ^6^ Shanghai Institute of Materia Medica Chinese Academy of Sciences Shanghai 201203 China

**Keywords:** cell–cell interaction, endometrial hyperplasia, endothelial cell activation, SHP2‐RIPK1‐AP‐1 axis, sterile inflammation

## Abstract

Sterile inflammation occurs in various chronic diseases due to many nonmicrobe factors. Examples include endometrial hyperplasia (EH), endometriosis, endometrial cancer, and breast cancer, which are all sterile inflammation diseases induced by estrogen imbalances. However, how estrogen‐induced sterile inflammation regulates EH remains unclear. Here, a single‐cell RNA‐Seq is used to show that SHP2 upregulation in endometrial endothelial cells promotes their inflammatory activation and subsequent transendothelial macrophage migration. Independent of the initial estrogen stimulation, IL1β and TNFα from macrophages then create a feedforward loop that enhances endothelial cell activation and IGF1 secretion. This endothelial cell–macrophage interaction sustains sterile endometrial inflammation and facilitates epithelial cell proliferation, even after estradiol withdrawal. The bulk RNA‐Seq results and phosphoproteomic analysis show that endothelial SHP2 mechanistically enhances RIPK1 activity by dephosphorylating RIPK1^Tyr380^. This event activates downstream activator protein 1 (AP‐1) and instigates the inflammation response. Furthermore, targeting SHP2 using SHP099 (an allosteric inhibitor) or endothelial‐specific SHP2 deletion alleviates endothelial cell activation, macrophage infiltration, and EH progression in mice. Collectively, the findings demonstrate that SHP2 mediates the transition of endothelial activation from estradiol‐driven acute inflammation to macrophage‐amplified chronic inflammation. Targeting sterile inflammation mediated by endothelial cell activation is a promising strategy for nonhormonal intervention in estrogen‐related diseases.

## Graphical abstract

SHP2 mediates the transition of endothelial activation from estradiol‐driven acute inflammation to macrophage‐amplified chronic inflammation. Activated endothelial cells interact with macrophages and epithelial cells to generate and sustain endometrial sterile inflammation, thus promoting endometrial hyperplasia by IGF1. Endothelial SHP2 mechanistically leads to the inflammatory activation of the endothelial cell through activating RIPK1‐AP‐1 axis after primary estrogen stimulation.

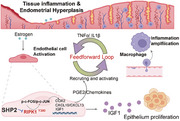

## 1 Introduction

Inflammation that occurs under sterile conditions is triggered by autoantigens that change in quantity, quality, structure, and localization. Changes in these antigens lead to a transition from self to non‐self, resulting in sterile inflammation.^[^ ^1^, ^2^, ^3^ ^]^ This transition is known to be a consequence of altered estrogen levels (increases or decreases); thus, sterile inflammation is a common symptom of many estrogen‐related diseases, such as breast cancer, cervical carcinoma, ovarian cancer, endometrial cancer, endometrial hyperplasia (EH), endometriosis, and bone diseases.^[^ ^4^, ^5^, ^6^, ^7^, ^8^, ^9^, ^10^ ^]^ However, apart from estrogen–estrogen receptor signaling, the actual signaling pathways that are regulated by estrogen are not well studied in these estrogen‐related diseases. This is particularly the case for EH, which is primarily driven by excess estrogen.^[^ ^11^ ^]^

The histological change in EH is an abnormal proliferation of endometrial epithelial cells, which leads to an elevated endometrial gland‐to‐stroma ratio and cytological atypia because of the irregular proliferation of the endometrial glandular epithelium.^[^ ^12^ ^]^ EH has a widely recognized involvement in reproductive failure, and if left untreated, ≈40% of EH cases can progress to endometrial cancer.^[^ ^13^, ^14^ ^]^ Progestin supplementation is the only nonsurgical management used to treat EH; however, some patients exhibit no response or show resistance to progestin therapy.^[^ ^15^ ^]^ Some studies have reported progestin side effects and even an increased risk of progression to carcinoma after progestin therapy.^[^ ^16^ ^]^ Therefore, studies are needed to establish the pathological mechanisms of EH and a nonhormone pharmacologic intervention to treat EH.

The abnormal epithelial cell proliferation observed in EH is regulated only in part by estrogen, as other factors, including cytokines and growth factors, also come into play.^[^ ^11^, ^17^ ^]^ The observation that infiltration of immune cells increases as the disease progresses through different stages^[^ ^11^, ^18^, ^19^ ^]^ suggests that tissue inflammation may be one of the regulators of EH. However, no clinical applications of anti‐inflammation drugs or strategies targeting molecules involved in inflammation have yet emerged as EH treatments.

It is widely recognized that almost all pathological inflammation events are associated with dysfunctions of endothelial cells, the most important regulators of organ‐specific immune response and tissue inflammation.^[^ ^20^ ^]^ The activation of endothelial cells increases blood flow, vascular permeability, and transendothelial leukocyte migration into tissues.^[^ ^21^, ^22^ ^]^ In addition, activated endothelial cells secrete extracellular proteins, including chemokines, cytokines, and growth factors.^[^ ^21^ ^]^ However, most studies on inflammation have focused mainly on immune cells and parenchymal cells, with less attention paid to endothelial cells. Consequently, knowledge of the mechanisms underlying endothelial cell activation of inflammation and targeted therapies to suppress this inflammation remains limited.

One potential target in many inflammatory diseases is SHP2, a tyrosine phosphatase encoded by *PTPN11* that regulates the phosphorylation of tyrosine on its substrate protein.^[^ ^23^, ^24^, ^25^, ^26^, ^27^ ^]^ Notably, the SHP2 hyperactivation observed in patients with Noonan syndrome (NS) is driven by the development of macrophage‐mediated meta‐inflammation and insulin resistance.^[^ ^26^ ^]^ Consistently, NS patients may also develop psoriasis,^[^ ^28^ ^]^ and we previously found an involvement of high levels of SHP2 in macrophages in psoriasis.^[^ ^27^ ^]^ Under these conditions, SHP2 hyperactivation is induced by gene mutation or inflammation signaling. However, no studies have reported a response of SHP2 to estrogen; therefore, its potential role in the hormone‐induced sterile inflammation seen in EH and any underlying mechanisms remains to be investigated.

At present, a phase III clinical trial of a SHP2 inhibitor, JAB‐3312, has been approved by the Center for Drug Evaluation in China.^[^ ^29^ ^]^ A function for SHP2 in human reproduction is also being revealed. Notably, SHP2 appears to be involved in female infertility^[^ ^30^ ^]^ and endometriosis,^[^ ^31^ ^]^ while also playing essential roles in the physiological function of embryo implantation and stromal decidualization.^[^ ^32^ ^]^ Collectively, these reports indicate that SHP2 expression may respond to estrogen and that SHP2 may have various functions in different estrogen‐related disorders. Thus, targeting cellular SHP2 functions using a specific inhibitor may be a promising nonhormone pharmacotherapy for EH diseases.

In this study, we show that SHP2 in endothelial cells senses excess estradiol stimulation and responds by driving the inflammatory activation of endothelial cells. This response is achieved through the dephosphorylation of RIPK1 at the Tyr380 site and the activation of transcription factor complex AP‐1, which promotes inflammatory gene transcription and macrophage infiltration. Thus, macrophage‐involved sterile inflammation amplifies the activation of endothelial cells, causing them to secrete greater amounts of IGF1, which then results in epithelial cell proliferation and EH progression. Collectively, our results support the importance of endothelial cell activation and continuation of estradiol‐independent sterile inflammation as regulators of EH.

## 2 Results

### 2.1 Endothelial SHP2 Levels are Abnormally Increased in EH and Associated with Pathogenesis

**Figure 1:**
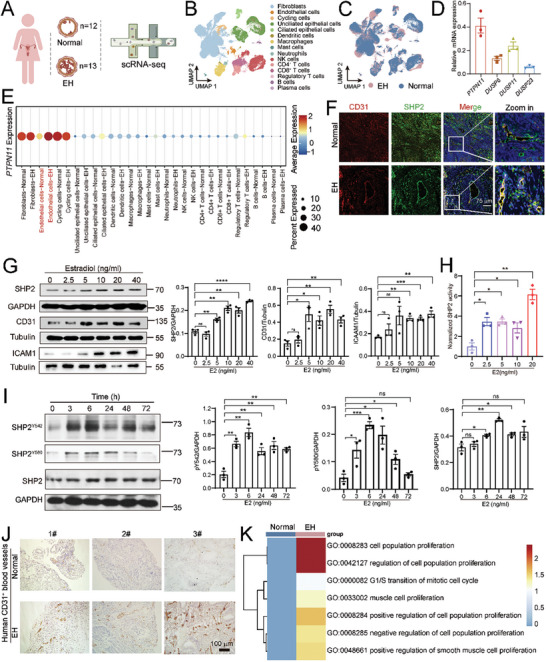
SHP2 in endothelial cells is upregulated and activated by estradiol (E2) in endometrial hyperplasia (EH) tissue. A) Schematic representation of clinical human endometrium sample collection for single‐cell RNA sequencing (scRNA‐Seq). Normal, normal endometrium, n = 12; EH, endometrium from patients with endometrial hyperplasia, n = 13. B) and C) The uniform manifold approximation and projection (UMAP) visualization showing all the cell clusters colored according to cell subsets (B) and samples (C). D) Relative mRNA expression of the four indicated genes in human umbilical vein endothelial cells (HUVECs). E) Dot plot showing the expression of *PTPN11* in different cell types. F) Expression of SHP2 (green) in CD31^+^(red) vessels from samples of normal and EH endometrium (n = 5). Scale bar, 100 µm. G) The protein expression of SHP2, CD31, and ICAM1 was examined and quantified in HUVECs following treatment with different doses of estradiol for 24 h. Dunnett *t*‐test. H) SHP2 enzyme activity was assessed in HUVECs after treatment with different doses of estradiol for 24 h. Dunnett *t*‐test, ^*^ *P* value <0.05; ^**^ *P* value <0.01 compared with the 0 group. I) Levels of phosphorylated SHP2 in HUVECs treated with 40 ng/mL estradiol for the indicated time. The protein expression was quantified. Dunnett *t*‐test. J) IHC staining of CD31^+^ vessels in normal and EH endometrium (n = 5). K) GO analysis of endothelial cells from the scRNA‐Seq data.

Our research has had a long‐established focus on the function of protein tyrosine phosphatases (PTPs) in diverse chronic inflammatory diseases.^[^ ^23^, ^33^ ^]^ Here, we investigated the role of PTPs in EH by first collecting 13 endometrium samples from EH patients and 12 normal endometrium samples for single‐cell RNA sequencing (scRNA‐Seq) to profile all the PTPs (**Figure** **1**A). We identified 15 clusters of cell types (Figure 1B,C). We studied the differences in PTP expression between normal and EH samples by analyzing all the PTPs detected in 15 cell types (Figure S1A, Supporting Information). Unexpectedly, a comparison of the normal and EH groups revealed a greater number of differentially expressed genes in endothelial cells than in epithelial cells. The *DUSP23*, *DUSP11*, *DUSP6*, and *PTPN11* genes showed high expression and significantly different expression between endothelial cells from the normal and EH groups (Figure S1B, Supporting Information).

We also found that *PTPN11*, which encodes SHP2, showed the highest mRNA expression among the four differentially expressed genes in endothelial cells (Figure 1D). In addition, the changes in *PTPN11* gene expression were observed specifically in endothelial cells but not in other cell types (Figure 1E). Thus, we hypothesized that the increased *PTPN11* expression in the endothelial cells of EH tissues may play a role in EH pathogenesis.

Immunofluorescence staining confirmed the increases in SHP2 in endometrial vessels from patients with EH (labeled with CD31, red) (Figure 1F). We then explored whether the increased SHP2 expression was directly regulated by estradiol by culturing human umbilical vein endothelial cells (HUVECs) with different doses of estradiol for 24 h. As expected, the expression of SHP2 in HUVECs was upregulated by estradiol (Figure 1G; Figure S1C, Supporting Information). The expression of CD31 and ICAM1, markers of endothelial cell activation, also increased after estradiol treatment (Figure 1G). The enzyme activity of SHP2 increased after estradiol stimulation (Figure 1H). The phosphorylation of SHP2 at Tyr542 and Tyr580, indicating SHP2 activation, also increased (Figure 1I). However, in agreement with the scRNA‐Seq results, SHP2 expression was not influenced by estradiol in human endometrial epithelial cells (hEECs). This might possibly reflect a lower basal SHP2 expression in hEECs (Figure S1D,E, Supporting Information).

EH often accompanies angiogenesis and abnormal bleeding as a result of endothelial cell dysfunction,^[^ ^34^, ^35^ ^]^ and we found increased numbers of endometrial vessels in EH samples (Figure 1J). Further GO analysis revealed an upregulation of the proliferation signaling pathway in endothelial cells (Figure 1K). Hence, our results identified increased SHP2 expression and enzyme activity in endothelial cells in response to excessive estradiol stimulation and a consequent vascular dysfunction in EH.

### 2.2 Endothelial Cell‐Specific Deletion or Inhibition of SHP2 Alleviates Estradiol‐Induced EH in Mice and in Human Endometrial Organoids

**Figure 2:**
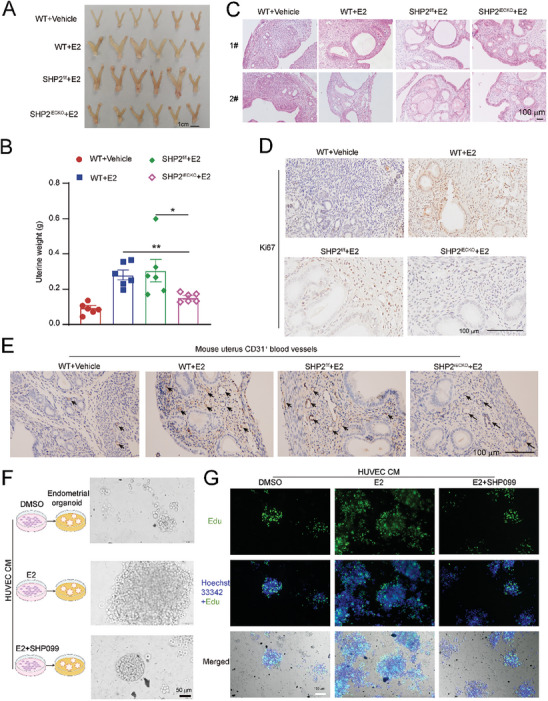
SHP2‐specific deletion or inhibition in endothelial cells reverses estradiol (E2)‐induced endometrial hyperplasia (EH) in mice and in a human EH organoid model. A) The morphology of the uteruses from 4 different groups of mice subjected to the indicated treatments (n = 6/group). B) The uterine weights of mice from the indicated groups (n = 6/group). Tukey‐Kramer test. C) Hematoxylin/eosin (H&E) staining of mouse uteruses from the 4 groups (n = 6/ group). D) Immunohistochemical (IHC) staining of Ki67^+^ proliferative cells in the mouse uteri from the 4 groups. E) IHC staining of CD31^+^ vessels in the mouse uteri from the four groups. The WT+ Vehicle group represents the wildtype (WT) C57BL/6 mice treated with olive oil for 21 days. The WT+E2 group represents the WT C57BL/6 mice treated with estradiol (100 µg kg^−1^) for 21 days. The SHP2^f/f^ +E2 group represents the *Shp2* ^flox/flox^ transgenic mice with C57BL/6 background treated with E2 (100 µg kg^−1^) for 21 days. The SHP2^iECKO^ +E2 group represents transgenic mice (C57BL/6 background) with SHP2 deletion specifically in the endothelial cells (SHP2^iECKO^) and treated with E2 (100 µg kg^−1^) for 21 days. F) HUVECs were treated with or without E2 (40 ng mL^−1^) and SHP099 (5 µM; an allosteric inhibitor of SHP2) for 24 h, and then in a refreshed medium for another 24 h. The conditional media (CM) from the different HUVEC treatments were collected and used to culture human EH organoids on day 3 after the organoid seeding for 7 days. A bright‐field image of organoids cultured with different CM showed the different sizes of organoids. G) Proliferating organoids after HUVEC CM culture for 3 days, followed by the addition of 20 µM EdU and incubation for 3 h. The green Edu‐positive foci indicated cell proliferation in the organoids.

We investigated the direct role of endothelial SHP2 in EH pathogenesis using inducible endothelial‐specific SHP2 knockout mice (SHP2^iECKO^, Figure S2A,B, Supporting Information). We constructed an EH model by exposing the mice to estradiol for 21 consecutive days. The treatment did not influence the mouse's body weight (Figure S2C, Supporting Information). SHP2 expression decreased in endothelial cells following 5 days of tamoxifen injections in SHP2^iECKO^ mice (Figure S2D–F, Supporting Information). Compared to the WT vehicle control mice, the uteruses of mice from the other three groups were markedly larger, whereas the uteruses from SHP2^iECKO^ mice were smaller than those from SHP2^f/f^ mice (**Figure** **2**A). The uterine weight was significantly lower for the SHP2^iECKO^ mice than for the SHP2^f/f^ mice (Figure 2B). Histological evaluation of the mouse uteruses after estradiol exposure revealed a proliferative endometrium with architectural abnormalities and an increased gland‐to‐stroma ratio in both the WT and SHP2^iECKO^ groups (Figure 2C,D). However, the histological changes in terms of EH were slight and moderated in the SHP2^iECKO^ mice (Figure 2C). We also observed an increase in the number of endometrial vessels in the WT and SHP2^f/f^ groups after estradiol exposure but a smaller increase in the SHP2^iECKO^ group (Figure 2E).

We also ensured a more comprehensive experimental verification by incorporating experiments on a preclinical organoid model to study the role of endothelial SHP2 in a more complex biological context. We confirmed that endometrial organoids reproduced the glandular epithelial structure of endometrial tissue (Figure S3A,B, Supporting Information). The results of immunofluorescence staining showed that endometrial tissue exhibited the estrogen receptor α (ERα) expression, which was retained in the endometrial organoids (Figure S3C,D, Supporting Information). Additionally, the EpCAM expression confirmed their epithelial nature (Figure S3C,D, Supporting Information). The genetic characterization of both primary endometrial tissue and constructed endometrial organoid were conducted using short tandem repeat (STR) sequencing. Organoids used in the experiments and their frozen primary endometrial tissue were collected for STR sequencing. The STR reports indicated that the endometrial organoids matched with their source endometrial tissues (Supporting material S1 and S2, Supporting Information). The endometrial organoids generated by the current methods usually consist only of endometrial epithelial cells and some fibroblasts but do not contain endothelial cells.^[^ ^36^, ^37^ ^]^ However, because SHP2 appeared to play a different role in endometrial endothelial cells after exposure to E2 and promoted endometrial epithelial cell growth, we treated the E2‐treated organoids with conditional medium (CM) from endothelial cells (HUVECs) or with the SHP2 allosteric inhibitor, SHP099 (Figure 2F). The results of the organoid‐based experiments showed that CM from E2‐treated endothelial cells enhanced the expansion of the endometrial organoid, but the effect was reversed by inhibiting SHP2 activity in endothelial cells by treatment with SHP099 (Figure 2F). The 5‐ethynyl‐2′‐deoxyuridine (EdU) incorporation assay also demonstrated that CM from E2‐treated endothelial cells promoted the proliferation of endometrial organoids and that SHP099 treatment weakened this proliferation (Figure 2G). Taken together, our data obtained using both models demonstrated a positive involvement of endothelial SHP2 in EH progression.

### 2.3 SHP2‐Activated Inflammatory Endothelial Cells Secrete More Extracellular Factors

**Figure 3:**
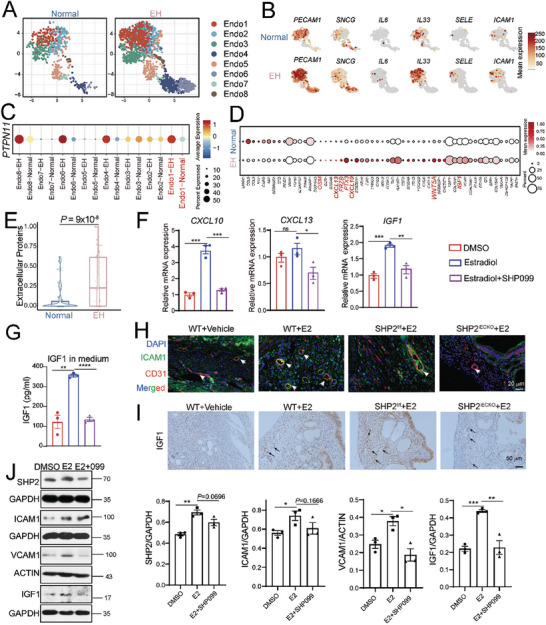
Estradiol (E2)‐triggered SHP2 expression promotes endothelial inflammatory activation and extracellular protein secretion. A) The UMAP plot shows different subtypes of endothelial cells, according to the scRNA‐Seq data. B) The signature gene markers of activated endothelial subtypes. C) Dot plot showing *PTPN11* expression in different endothelial cell subtypes. D) Dot plot showing the differentially expressed genes for extracellular proteins secreted from activated endothelial cells. E) Statistical analysis of all extracellular protein levels in endothelial cells. Student's *t*‐test. F) Validation of the gene expression of the indicated extracellular proteins in HUVECs by qPCR after stimulation with 40 ng mL^−1^ E2 in the presence or absence of SHP099 (5 µM) for 24 h. Dunnett *t*‐test. G) IGF1 levels in culture medium detected by ELISA after stimulation of HUVECs with 40 ng mL^−1^ E2 with or without SHP099 (5 µM) for 48 h. Dunnett's *t*‐test. H) Inflammatory activation of endothelial cells (CD31^+^ICAM1^+^) in mouse uteruses from the indicated groups (n = 6/group). I) IGF1 expression in mouse uteruses from the four indicated groups (n = 6/group). J) Western blot analysis of the proteins associated with endothelial cell inflammatory activation in HUVECs treated with E2 or SHP099 for 24 h. The protein expression is quantified. Dunnett's *t* test. ^*^ *P* value < 0.05; ^**^ *P* value < 0.01; ^***^ *P* value < 0.001.

Endothelial cells can respond to estrogen^[^ ^38^ ^]^ and transform from the resting state to the activated state.^[^ ^21^ ^]^ Estrogen activates G‑protein‐coupled receptors (GPCRs) to induce type I activation of endothelial cells, which is typically followed by type II activation.^[^ ^21^ ^]^ We characterized molecular and functional heterogeneity at the single‐cell level in endometrial endothelium cells from healthy individuals and patients with EH. Subtyping the endothelial cells into eight clusters (**Figure** **3**A) revealed that the endo1 subtype expressed high levels of inflammatory cytokines and leukocyte adhesion molecules, such as ICAM1, IL33, IL6, SELE, and SNCG (Figure 3B). Interestingly, the inflammatory endothelial cell cluster 1 subtype from EH samples also had increased *PTPN11* expression (Figure 3C).

We also measured the Ca^2+^ level after E2 stimulation, given that an increased Ca^2+^ level is an early step in endothelial activation. A rise in cytosolic Ca^2+^ triggers a plethora of cellular responses, including cell activation, secretion, and immunity. We found that estradiol treatment quickly increased intracellular Ca^2+^; however, pretreatment with SHP099 did not influence the Ca^2+^ influx in HUVECs, indicating that the early events occurring during type I endothelial cell activation are not dependent on SHP2 (Figure S4A‐C  Supporting Information). However, because the increases in intracellular Ca^2+^ concentration could occur by Ca^2+^ influx or by Ca^2+^ release from intracellular Ca^2+^ stores, we also determined whether E2 stimulation induced Ca^2+^ release from intracellular Ca^2+^ stores in HUVECs. We first used EGTA to chelate the Ca^2+^ in the assay medium before the E2 treatment. We then added 2 mM CaCl_2_ back to the medium to induce Ca^2+^ influx. We found that E2 stimulation quickly induced intracellular Ca^2+^ release, with a subsequent Ca^2+^ influx from outside the endothelial cells (Figure S4D,E, Supporting Information).

These results were consistent with those of previous studies showing that endothelial cell activation was usually triggered by the binding of a ligand (including E2) to the extracellular domains of heterotrimeric GPCRs. The exchange of guanosine diphosphate for guanosine triphosphateon the heterotrimeric G‐protein αq subunit of the receptor‐associated heterotrimeric G protein transmits a signal downward that promotes the release of the αq subunit from the G‐protein βγ dimer and activates the β isoforms of phospholipase C (PLCβ), which catalyzes the release of inositol‑1,4,5‐trisphosphate (InsP3) from phosphatidylinositol‑4,5‐bisphosphate (PtdIns(4,5)P2) from membrane lipids.^[^ ^39^ ^]^ In endothelial cells, InsP3 then induces elevations in cytosolic free Ca^2+^ by release from endoplasmic reticulum Ca^2+^ stores—these rises may take the form of oscillatory, transient changes in Ca^2+^ levels or an elevated plateau of cytosolic Ca^2+^. The cytosolic Ca^2+^ increases occurring after E2 stimulation may then be sustained by the entry of extracellular Ca^2+^.^[^ ^40^, ^41^ ^]^ Therefore, E2 appeared to promote rapid endothelial cell activation that could be sustained through Ca^2+^ influx, and SHP2 was involved in long‐term E2‐induced endothelial cell activation.

We observed that SHP2 overexpression increased the expression of ICAM1 and COX2, which are markers of endothelial cell activation (Figure S4F,G, Supporting Information). Therefore, these results indicated that SHP2 was a molecule involved in the inflammatory activation of endothelial cells. Recognizing that activated endothelial cells would release extracellular proteins to remodel the tissue microenvironment, we used scRNA‐Seq to analyze the extracellular proteins and detected significant increases in proteins secreted from endothelial cells from patients with EH (Figure 3D,E, Supporting Information). We also detected activated endothelial cells in the endometrial tissue samples from patients with EH (CD31^+^ICAM1^+^, Figure S4H, Supporting Information).

To test whether the extracellular proteins found in scRNA‐Seq data were induced by estradiol, we further picked up the extracellular proteins closely associated with inflammation and proliferation from the results of scRNA‐Seq and detected them in HUVECs after estradiol stimulation in vitro (Figure 3F,G; Figure S4I, Supporting Information). E2 increased the levels of CXCL10, CXCL13, and IGF1 in the activated HUVECs (Figure 3F,G), but these increases were reversed when SHP2 was inhibited with SHP099 (Figure 3F,G). We also confirmed endothelial activation in the mouse EH model by immunofluorescence staining, which showed higher numbers of activated endothelial cells (CD31^+^ ICAM1^+^) in EH mice than in vehicle control and SHP2^iECKO^ mice (Figure 3H). IGF1 expression, and especially IGF1 staining around blood vessels, both increased in EH mouse uterus samples (Figure 3I), whereas IGF1 expression decreased in the uterus of SHP2^iECKO^ mice, suggesting a positive regulation of IGF1 by endothelial SHP2 (Figure 3I). In vitro, the SHP2‐mediated endothelial inflammatory activation in HUVECs was indicated by high levels of ICAM1 and VCAM1 after estradiol stimulation (Figure 3J). Taken together, our data suggest that SHP2 triggered endothelial activation and promoted CXCL10, CXCL13, and IGF1 secretion after estradiol stimulation.

### 2.4 SHP2‐Triggered Endothelial Cell Activation Generates and Sustains Estradiol‐Independent Sterile Inflammation in EH

**Figure 4:**
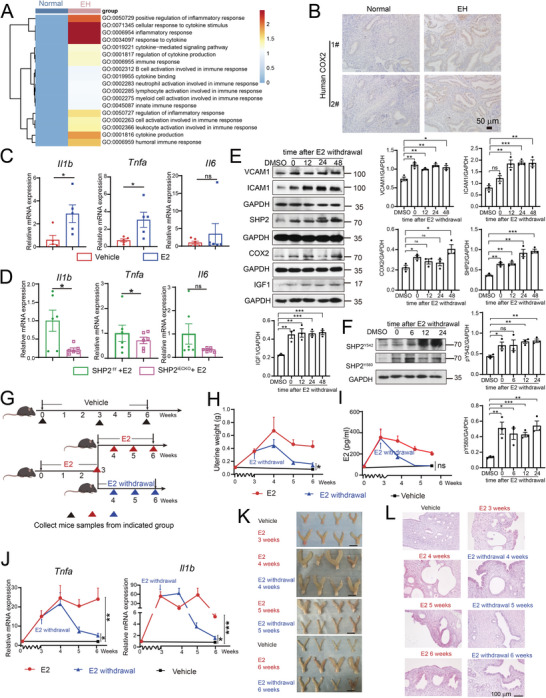
Endothelial activation generates and sustains long‐term sterile inflammation in endometrial hyperplasia (EH). A) GO analysis of the differentially expressed genes in endothelial cells revealed by scRNA‐Seq data from healthy and EH endometriums. B) Immunohistochemical staining of COX2 expression in human endometrial tissue. n = 5. C) The mRNA expression of *Il1b*, *Tnfa*, and *Il6* in mouse uteri treated with estradiol (E2) or vehicle in WT or D) or SHP2^f/f^ or SHP2^iECKO^ mice (n = 5 in the WT group, n = 6 in the SHP2^f/f^ group, and n = 6 in the SHP2^iECKO^ group). E) Western blot analysis of the proteins indicated endothelial cell inflammatory activation in HUVECs treated with E2 for 24 h and in HUVECs in which E2 was withdrawn at the indicated times. Protein expression was quantified and statistically analyzed as indicated to the right of and below the bands. Dunnett's *t*‐test. F) Western blot analysis of SHP2 phosphorylation after E2 treatment of HUVECs for 3 h and then withdrawal of E2 for the indicated time. The protein expression was quantified and statistically analyzed as indicated to the right of and below the bands. Dunnett's *t*‐test. G) Design of the animal experiment used to determine EH tissue inflammation after E2 withdrawal. Mouse uteri were first collected at 3 weeks to construct the EH model. Mice from the E2‐treated group were then divided into E2 and E2‐withdrawal groups. The E2‐withdrawal group was left untreated for another three weeks, while the mice from the E2 group were treated with E2 for a total of six weeks. H) Uterine weights from the indicated groups. I) E2 concentrations determined by ELISA in sera from mice of the indicated groups. J) The mRNA expression of *Tnfa* and *Il1b* in mouse uteruses at different time points. K) The morphology of mouse uteruses from the different groups at the indicated time points. Scale bar: 1 cm. L) H&E staining of mouse uterine tissues from the different groups at the indicated time points. Data represent the mean ± SEM. ^*^ *P* value < 0.05; ^**^ *P* value < 0.01, ^***^ *P* value < 0.001; ns indicates no significant difference.

The activation of vascular endothelial cells is involved in tissue sterile inflammation.^[^ ^42^ ^]^ Previous studies have reported that the numbers of neutrophils and T cells are significantly increased in EH patients.^[^ ^43^ ^]^ In addition to being driven by estrogen, endometrial proliferation can be promoted by cytokines and growth factors.^[^ ^18^ ^]^ Therefore, we proposed that endothelial cell activation generated an inflammatory endometrial environment that advanced the progression of EH. Based on this hypothesis, we first identified the inflammatory GO pathways that were enriched in EH endothelial cells (**Figure** **4**A). Consistently, COX2 showed increases in the endometria of patients with EH and EH mice (Figure 4B; Figure S5A, Supporting Information). *Il1β* and *Tnfα* gene expression was also significantly increased in the uterine tissues of EH mice (Figure 4C,D), whereas *Il6* expression showed no significant change. The levels of these inflammatory cytokines were lower in the uteruses of SHP2^iECKO^ mice than in SHP2^f/f^ mice (Figure 4C,D), indicating that estradiol‐induced uterine inflammation was mediated by endothelial SHP2. Collectively, these results confirmed the presence of sterile inflammation in the uteri of EH patients and EH mice and that uterine inflammation was regulated by SHP2 in endothelial cells.

This finding raised the question of whether and how endothelial cell activation could be sustained for prolonged periods, as estrogen levels are not always high in EH diseases. Therefore, we treated HUVECs with estradiol for 48 h and then withdrew estradiol at various intervals. This treatment resulted in sustained expression and activation of SHP2, as well as maintenance of high levels of ICAM1, VCAM1, COX2, and IGF1 (Figure 4E,F). We confirmed that long‐term endothelial cell activation and sterile inflammation did not require the continuous presence of estrogen in vivo by constructing an EH model by injecting mice for three weeks with estradiol and then withdrawing 20 mice from the estradiol treatment for another one to three weeks, while the remaining mice (EH group) continued to be injected with estradiol (Figure 4G). We collected the uteruses and serum from selected mice at different time points to observe the progression of EH and tissue inflammation (Figure 4H–L). The mouse uterine weights and sizes increased over time in the group subjected to six weeks of continuous estradiol (Figure 4H,K). By contrast, although the serum estradiol levels quickly decreased and recovered to baseline after two weeks of estradiol withdrawal in the estrogen withdrawal group (at the time point of five weeks) (Figure 4I), the uterine weights and sizes in this group also continued to increase even after one week of no E2 injections (i.e., at the four‐week time point), and then declined after two or three weeks of estradiol withdrawal (at the five weeks and six weeks time points). Interestingly, the absolute weights and sizes of the mouse uteri were still significantly higher in the E2 withdrawal group than in the vehicle control group (Figure 4H,K). The endometrium still displayed the EH phenotype and did not recover to the normal state.

In line with these morphological changes, the tissues showed persistent, sustained inflammation and histological changes (Figure 4J,L; Figure S5B, Supporting Information). Furthermore, the expression of tissue IGF1 and endothelial SHP2 (CD31^+^ SHP2^+^ endothelial cells) remained high in the EH samples even after estradiol withdrawal (Figure S5C,D, Supporting Information). Collectively, these results indicated that after the construction of the EH model at three weeks, the tissue inflammation environment and endothelial cell activation were sustained in the mouse uterus and persisted even after the estradiol amounts declined to normal levels. In summary, the SHP2‐mediated endothelial cell activation generated and sustained sterile inflammation in the uteri of EH mice. Endometrium inflammation was also involved in EH pathology through the promotion of IGF1 secretion. Continuous exposure to estradiol was therefore not necessary for long‐term sterile inflammation, but it served as the driver of endometrium inflammation and EH.

### 2.5 Inflammatory Endothelial Cells Sequentially Traffic and Activate Macrophages to Amplify Inflammation

**Figure 5:**
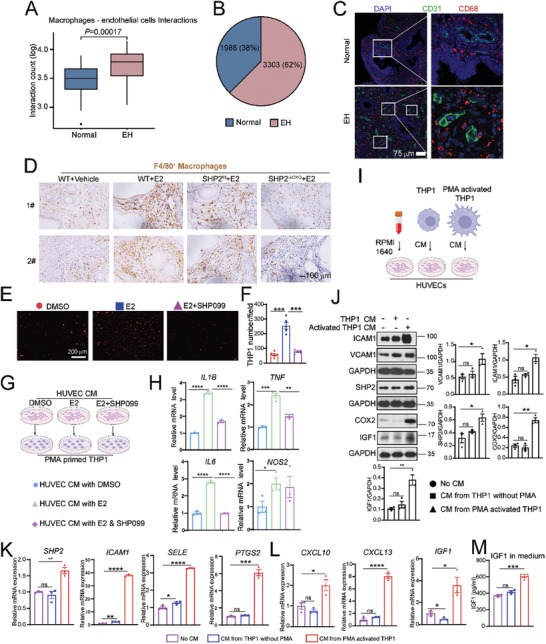
Endothelial cell–macrophage crosstalk undergoes positive feedback to enhance endometrial sterile inflammation. A) Interactions between macrophages and vascular endothelial cells predicted by CellPhoneDB. B) Pie chart shows the cell numbers and percentages of macrophages in the normal and EH patient groups. C) Immunofluorescence analysis of endothelial cells (CD31, green) and macrophages (CD68, red) in the endometrium from normal or patients with EH. D) Immunohistochemical staining of F4/80^+^ macrophages in mouse uterus tissues treated with estradiol (E2) or vehicle in WT, SHP2^f/f^, or SHP2^iECKO^ mice. E) The attachment of Dil‐labeled THP1 cells to HUVECs after E2 treatment (40 ng mL^−1^) with or without SHP099 (5 µM) for 24 h. F) Statistical analysis of the number of attached THP1 cells in five fields of one representative experiment. G) The experimental design used for the cell cultures is shown in Figure 5H. HUVECs were treated with E2 (40 ng mL^−1^) or SHP099 (5 µM) for 24 h and then incubated in a fresh medium for another 24 h. The HUVEC medium was then collected to culture the THP1 cells. H) The qPCR detection of mRNA levels for *IL1B*, *TNF*, *IL6*, and *NOS2* in THP1 cells cultured with conditional medium (CM) obtained from HUVECs pretreated with dimethylsulfoxide (DMSO) or estradiol (E2, 40 ng mL^−1^) with or without SHP099 (5 µM) for 48 h. I) Experimental outline of the different treatments used on the indicated cells. The CM from THP1 cells treated with or without PMA (100 ng mL^−1^) for 24 h was collected and replaced with complete RPMI 1640 medium for another 24 h of culture. The CM from the THP1 cells or complete RPMI 1640 were then used to treat HUVECs for 24 h. J) Western blot analysis of proteins indicated endothelial inflammatory activation after treatment of HUVECs with CM from THP1 cells. The protein expression was quantified and analyzed as shown to the right of and below the bands. K) The qPCR detection of mRNA levels of SHP2 and the genes indicated endothelial cell inflammatory activation after treatment with CM from THP1 cells. L) The qPCR detection of mRNA levels of genes encoding secreted extracellular proteins in activated HUVECs after treatment with CM from THP1 cells. M) IGF1 secretion into HUVEC medium after treatment with CM from THP1 cells. Data are presented as mean ± SEM. Data were analyzed using the Tukey‐Kramer test. ^*^ *P* value < 0.05; ^**^ *P* value < 0.01; ^***^ *P* value < 0.001; ns represents no significant difference.

Almost all the pathological events of inflammation are associated with the dysfunction of endothelial cells caused by the transendothelial migration of leukocytes.^[^ ^22^ ^]^ Therefore, we examined the types of immune cells being trafficked by endothelial cells into the EH endometrium. So, we used scRNA‐seq data analysis to characterize the network of interactions between endothelial cells and immune cells (Figure S6A,B, Supporting Information). The interactions between endothelial cells and macrophages were significantly enhanced in the EH samples (**Figure** **5**A), and the numbers and proportions of endometrial macrophages were higher in the EH samples, accounting for 62% (Figure 5B). We observed substantial numbers of infiltrating macrophages (CD68^+^) frequently localized close to CD31^+^ blood vessels (Figure 5C) in the endometrial tissue of patients with EH. In line with these findings, the IHC staining of F4/80^+^ macrophages was increased in EH mouse uteruses (Figure 5D; Figure S6C, Supporting Information) and decreased in SHP2^iECKO^ uteruses, suggesting that macrophage infiltration was dependent on endothelial SHP2 expression (Figure 5D).

We further addressed the interaction between endothelial cells and macrophages by first treating HUVECs with E2 or SHP099 for 48 h and then replacing the medium to co‐culture the HUVECs and THP1 cells for another 1 h. The estradiol‐activated endothelial cells effectively increased the adhesion of the THP1 cells to HUVECs (Figure 5E,F). Contrary to our hypothesis, E2 could not directly induce THP1 activation (Figure S6D, Supporting Information), but the conditional medium (CM) from E2‐treated endothelial cells promoted macrophage activation and the expression of IL1β, TNF, and IL6 (Figure 5G,H). By contrast, SHP099 treatment reversed the effect of CM from estradiol‐cultured HUVECs on macrophage activation. We also used the CM from SHP2‐overexpressing HUVECs to treat THP1 cells and observed a stronger effect on macrophage activation than was achieved using CM from estradiol‐treated HUVECs (Figure S6E, Supporting Information). Taken together, these results demonstrated that high expression of SHP2 induced by estradiol promoted endothelial activation, which then attracted and activated macrophages and ultimately generated an inflammatory endometrium environment.

We also cultured HUVECs with CM from THP1 cells pretreated with or without activation with phorbol 12‐myristate 13‐acetate (PMA; 100 ng mL^−1^) for 24 h (Figure 5I). The CM from activated macrophages upregulated the protein expression of SHP2, ICAM1, VCAM1, COX2, and IGF1 (Figure 5J). Consistent with the increased protein level, the mRNA levels also increased for *PTPN11, PTGS2*, and the adherens and junction molecules, including *ICAM1* and *SELE* (Figure 5K). The inflammatory cytokines and chemokines, including *CXCL10* and *CXCL13*, also increased in HUVECs after treatment with CM from activated THP1 cells (Figure 5L). The mRNA and protein levels of IGF1 were significantly increased in HUVECs treated with CM from activated THP1 cells (Figure 5L,M). To mimic the function of HUVEC CM in activated macrophages, we stimulated HUVECs with estradiol or with different inflammatory cytokines. The effects on SHP2 expression and endothelial activation were stronger after stimulation with inflammatory factors than after estradiol stimulation alone (Figure S6F, Supporting Information). Together, these data revealed that excessive estradiol levels initially activated endothelial cells by upregulating SHP2, but then the activated endothelial cells facilitated tissue macrophage infiltration and activation. The cell–cell interactions therefore reshaped the inflammatory uterine environment through a feedforward loop. The activated macrophages further amplified endothelial inflammatory activation through cytokines, such as IL1β and TNFα, to sustain sterile endometrial inflammation and facilitate further secretion of IGF1.

### 2.6 IGF1 Derived from Activated Endothelial Cells Supports Endometrial Epithelial Cell Proliferation

**Figure 6:**
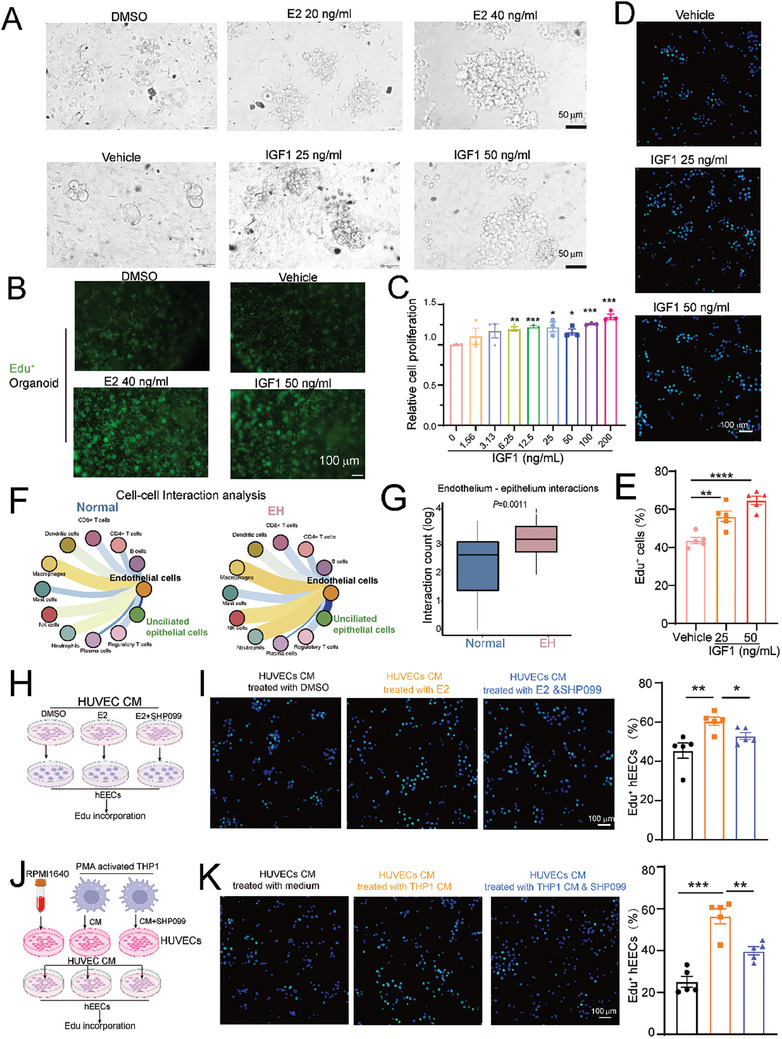
Increased IGF1 secretion in response to endothelial cell inflammatory activation promotes endometrial epithelial cell proliferation. A) A bright field image of human EH organoids showed the different sizes of organoids treated with estradiol (E2) or IGF1 on day 3 after organoid seeding and culturing for another 7 days. B) Organoids were seeded in 96‐well plate for 3 days, then treated with various CM for another 3 days. The proliferation was detected by adding 20 µM EdU to the medium and culturing for 3 h to detect EdU‐positive organoids. The green Edu‐positive foci indicated cell proliferation in the organoids. C) CCK8 detection of hEEC proliferation after treatment with different doses of recombinant IGF1 for 48 h. Dunnett's *t*‐test. D) EdU incorporation after IGF1 stimulation for 6 h, followed by the addition of 20 µM EdU to the medium and further culture for 3 h. E) The number of EdU^+^ hEECs after stimulation with different doses of IGF1 (n = 5). F) CellPhoneDB analysis of cell–cell interactions between endothelial cells, immune cells, and epithelial cells using scRNA‐seq data from normal and EH endometrium samples. G) Statistical analysis of the cell interactions between endothelial and epithelial cells. Student's *t*‐test. H) Schematic representation of HUVEC and hEEC co‐cultures. HUVECs were first pretreated with dimethylsulfoxide (DMSO) or estradiol (E2, 40 ng mL^−1^) with or without SHP099 (5 µM) for 24 h. The medium was then replaced with a complete Endothelial Cell Medium (ECM) and cultured for another 24 h. The conditional medium (CM) was collected and used to culture hEECs for 12 h. The hEECs were then subjected to EdU incorporation assays. I) The number of EdU^+^ hEECs after stimulation with different doses of IGF1 (n = 5). The statistical analysis is depicted on the right. Dunnett's *t*‐test. J) Schematic representation of co‐culturing HUVECs with THP1 cells: The THP1 cells treated with or without PMA (100 ng mL^−1^) were cultured for 24 h, the CM was collected and replaced with complete RPMI 1640 medium, and the cells were cultured for another 24 h. The CMs from the THP1 or complete RPMI 1640 treatments were used to activate the HUVECs for 24 h. The CMs from the activated HUVECs were then used to culture hEECs for 12 h for EdU incorporation assays. K) The number of EdU^+^ hEECs after the indicated treatments (n = 5). The statistical analysis is shown on the right. Dunnett's *t*‐test. ^*^ *P* value < 0.05; ^**^ *P* value < 0.01; ^***^ *P* value < 0.001; ^****^ *P* value < 0.0001.

The pathologic mechanisms underlying EH include the abnormal proliferation of endometrial epithelial cells. Our data showed that endothelial cells secreted large amounts of IGF1; therefore, our next aim was to determine the effects of IGF1 secreted by activated HUVECs on the proliferation of human endometrial epithelial cells (hEECs) and human endometrial organoids. As expected, estradiol treatment promoted the proliferation and expansion of human endometrial organoids (**Figure** **6**A,B). However, estradiol had no effect on hEEC cell cycle progression (Figure S7A,B, Supporting Information). This observation suggested that excess estradiol alone was not sufficient to initiate the proliferation of this human epithelial cell line and that other complex biological factors in organoids or tissues interacted with epithelial cells and acted synergistically with estrogen to promote abnormal epithelial proliferation.

We found that endothelial cells secreted increased amounts of IGF1 after activation, but the contribution of this paracrine secretion to the abnormal proliferation of epithelial cells was unclear. We demonstrated that IGF1 promoted a dose‐dependent proliferation of both hEECs and human endometrial organoids (Figure 6A–C), and the EdU assays further confirmed the positive effect of IGF1 on the proliferation of hEECs and human endometrial organoids (Figure 6B,D). These findings support a potential interaction between epithelial cells and endothelial cells through IGF1 signaling, which leads to abnormal epithelial cell proliferation. We also observed a significant enhancement of this interaction between endothelial and epithelial cells, as shown by the bolder blue line that connected the endothelial and epithelial cells in our scRNA‐Seq data (Figure 6F,G). We verified this enhanced interaction by culturing hEECs for 48 h with the CM from HUVECs pretreated with estradiol or with the CM from THP1 cells. The EdU assay demonstrated that the CM from estradiol‐activated or THP1‐activated HUVECs significantly promoted the proliferation of hEECs (Figure 6H–K). Consistently, the results of CCK8 assays also showed that the CM from HUVECs had a similar effect on the proliferation of hEECs (Figure S7C,D, Supporting Information). Overall, activated endothelial cells promoted abnormal epithelial cell proliferation through a paracrine secretion pathway, with one of the effector factors being the increased secretion of IGF1 from endothelial cells.

### 2.7 SHP2 Promotes Inflammatory Activation of Endothelial Cells Through AP‐1 Activation

**Figure 7:**
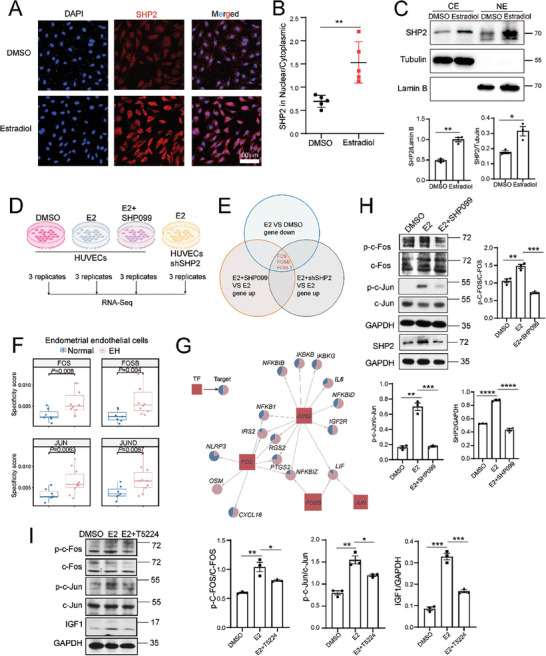
SHP2 promotes the transcription of IGF1 and inflammatory factors in endothelial cells by activating AP‐1. A) HUVECs were treated with estradiol (E2; 40 ng mL^−1^) for 48 h, and the SHP2 expression was detected by immunofluorescence staining. B) Quantification of the nuclear and cytoplasmic expression ratio was determined using GraphPad Prism 8.0 (n = 5). Student's *t*‐test. C) Western blot analysis of the expression of SHP2 in the nuclear and cytoplasmic compartments after estradiol (E2, 40 ng mL^−1^) treatment for 24 h. Student's *t*‐test. D) Study design of bulk RNA‐Seq analysis (n = 3/group). HUVECs were treated with dimethylsulfoxide (DMSO), estradiol, or estradiol plus SHP099 for 6 h, and SHP2‐knockdown HUVECs were treated with estradiol for 6 h. All cells were then subjected to RNA‐Seq (n = 3). E) The intersection of differentially expressed genes in HUVECs between two comparable groups treated as indicated. F) Transcription factor activities. The dot in the box plot represents the activity of the indicated transcription factor in each sample. Student's *t*‐test. G) Gene regulatory network of transcription factors and corresponding genes. The pie charts show the differential expression of the genes regulated by the indicated gene in normal and EH samples. H) Western blot analysis of AP‐1 activation after E2 treatment with or without SHP099 (5 µM) for 12 h. The protein expression was quantified and statistically analyzed. Dunnett's *t‐*test. I) WB analysis of AP‐1 activation and IGF1 expression after E2 treatment with or without the AP‐1 inhibitor T5224 pretreated for 3 h. The protein expression was quantified and statistically analyzed. Dunnett's *t‐*test. ^*^ *P* value < 0.05; ^**^ *P* value < 0.01; ^***^ *P* value < 0.001; ^****^ *P* value < 0.0001.

The ratio of SHP2 in the nucleus increased after the culture of HUVECs with estradiol for 48 h (**Figure** **7**A,B). Separation of the cytoplasmic and nuclear proteins revealed increases in SHP2 in both the cytoplasm and the nucleus (Figure 7C). We investigated the mechanisms of these estradiol‐triggered increases in SHP2 in terms of endothelial cell activation by conducting bulk RNA‐seq analysis of HUVECs treated with DMSO or estradiol with or without SHP099 and SHP2‐knockdown HUVECs treated with estradiol (Figure 7D; Figure S8A, Supporting Information). The cells treated with estradiol alone and SHP099 plus estradiol showed inverse expression patterns (Figure S8B–E, Supporting Information); for example, FOS and FOSB mRNA expression was upregulated after estradiol treatment, and these changes in mRNA expression were reversed by inhibiting SHP2 by SHP099 or SHP2 knockdown (Figure S8B–E, Supporting Information; Figure 7E). FOS and FOSB are members of the FOS family and form the AP‐1 transcription factor complex with c‐Jun. FOS, FOSB, JUN, and JUND were all detected in our scRNA‐Seq data from endothelial cells, and further transcription factor activity analysis confirmed the increase in transcription factor activity in endothelial cells from EH samples (Figure 7F). Target gene analysis of the four transcription factors showed increasement in cytokines, chemokines, and the members of the NF‐kappa B pathway in the endothelial cells from the EH samples, indicating an involvement in the inflammatory activation of endothelial cells (Figure 7G). Moreover, the genes involved in IGF1 signaling and metabolism, such as IRS2, IGS2, and IGF2R, were all upregulated in the EH samples (Figure 7G). We also found that estradiol promoted and SHP099 inhibited the phosphorylation of c‐FOS and c‐JUN (Figure 7H), indicating a role for phosphorylation, as the AP‐1 transcription factor requires phosphorylation for activation. This possibility was supported by the finding that the increase in IGF1 expression induced by estradiol was reversed by T5224, an inhibitor of the AP‐1 transcription factor (Figure 7I). T5224 treatment also decreased the mRNA expression of IGF1, ICAM1, SELE, and COX2 (Figure S8F, Supporting Information). Taken together, these results indicated that SHP2 regulated the phosphorylation of transcription factor AP‐1 and that AP‐1 positively regulated inflammatory gene transcription and, thus, inflammatory activation of endothelial cells.

### 2.8 SHP2 Enhances RIPK1 Activity by Dephosphorylating RIPK1 at the Tyr380 Site to Activate AP‐1

**Figure 8:**
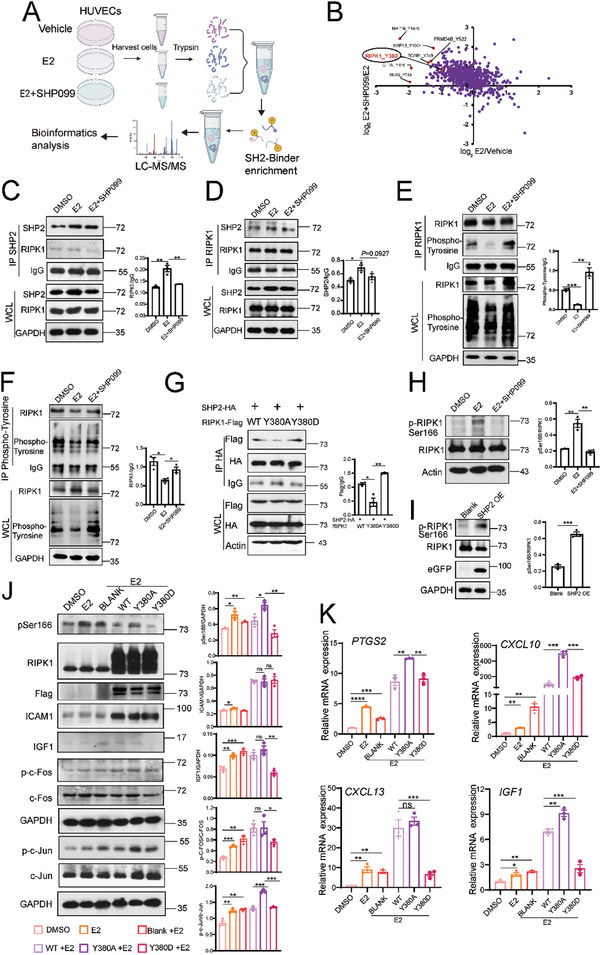
SHP2 binds to and dephosphorylates RIPK1^Tyr380^ to mediate the activation of AP‐1. A) Experimental outline for stable isotope labeling with amino acids for cell culture‐based tyrosine phosphoproteomics in HUVECs treated with vehicle, estradiol (E2; 40 ng mL^−1^), or E2 (40 ng mL^−1^) plus SHP099 (5 µM). n = 3/group. B) Quadrant diagram for tyrosine phosphoproteomic analysis. C) and D) Immunoprecipitation of HUVECs pretreated with SHP099 (5 µM) for 3 h and then treated with E2 (40 ng mL^−1^) for another 6 h. Cell lysates were immunoprecipitated using anti‐SHP2 (C) or anti‐RIPK1 antibodies (D). The RIPK1 protein was pulled down by anti‐SHP2 antibody, and the SHP2 protein was pulled down by anti‐RIPK1 antibody; both were quantified. Dunnett's *t‐*test. E) and F) Immunoprecipitation of HUVECs pretreated with SHP099 (5 µM) for 3 h and then treated with E2 (40 ng/mL) for another 6 h. Cell lysates were harvested and immunoprecipitated using (E) anti‐RIPK1 antibody or (F) anti‐phosphotyrosine antibody for immunoblotting with the indicated antibodies. (WCL; whole cell lysate.) The phosphotyrosine protein was immunoprecipitated by anti‐RIPK1 antibody, and the RIPK1 protein was immunoprecipitated by anti‐phospho‐tyrosine antibody; both are quantified. Dunnett's *t‐*test. G) Human embryonic kidney HEK293T cells were transfected with SHP2‐HA plasmid and WT‐RIPK1 or RIPK1^Y380A^ or RIPK1^Y380D^ point mutant plasmids for 48 h. The cells were then harvested and immunoprecipitated using anti‐HA antibody. The Flag protein pulled down by anti‐HA antibody is quantified. Dunnett's *t‐*test. H) Western blot analysis of the activation of RIPK1 (represented by p‐Ser166) after treatment with E2 or with E2 plus SHP099. The protein level of p‐RIPK1 phosphorylated at the Ser166 site is quantified. Dunnett's *t‐*test. I) Western blot analysis of the activation of RIPK1 after SHP2 overexpression. The protein level of p‐RIPK1 phosphorylated at the Ser166 site was quantified. Student's *t‐*test. J) Western blot analysis of endothelial cell activation‐related proteins and IGF1 expression after overexpression of RIPK1‐WT, RIPK1‐Y380A, or RIPK1‐Y380D in HUVECs. The protein expression was quantified. Dunnett's *t‐*test. K) Gene expression of endothelial cell activation after overexpression of RIPK1‐WT, RIPK1‐Y380A, or RIPK1‐Y380D in HUVECs. ^*^ *P* value < 0.05; ^**^ *P* value < 0.01; ^***^ *P* value < 0.001; ^****^ *P* value < 0.0001.

We determined the protein dephosphorylated by SHP2 using quantitative tyrosine phosphoproteomic analysis and stable isotope labeling with amino acids in cell culture (SILAC) (**Figure** **8**A). A total of 1329 phospho‐Tyr sites were identified, and 934 quantified phospho‐Tyr sites were included (Figure S9A, Supporting Information). SHP2 is known to dephosphorylate its substrate protein, and we found significant downregulation of phosphorylation in the estradiol‐treated group compared to the DMSO‐treated group, as well as significant upregulation in the group treated with estradiol plus SHP099 compared with the estradiol‐only group (Figure S9B–G, Supporting Information).

Our search to identify a protein involved in endothelial inflammatory activation and protein phosphorylation^[^ ^44^ ^]^ drew our attention to a protein kinase receptor‐interacting protein kinase 1 (RIPK1^Tyr380^), a key regulator of inflammation.^[^ ^45^, ^46^ ^]^ The phosphorylation of RIPK1^Tyr380^ was decreased after estradiol treatment but was increased by co‐treatment with estradiol and SHP099 (Figure 8B). Therefore, we viewed RIPK1 as a newly found SHP2 substrate that may be responsible for the phosphorylation of c‐FOS and c‐JUN.

Our coimmunoprecipitation assays revealed an endogenous interaction of SHP2 and RIPK1 and an enhancement of this interaction by estradiol treatment in HUVECs. However, SHP099 treatment disrupted this interaction (Figure 8C,D). Similarly, tyrosine phosphorylation of RIPK1 was decreased by estradiol treatment but was enhanced by the SHP099 treatment (Figure 8E,F). All of these results indicated that estradiol enhanced the interaction between SHP2 and RIPK1 and decreased the tyrosine phosphorylation of RIPK1.

We verified the specific tyrosine site and the kinase function of RIPK1^Tyr380^ by constructing two RIPK1 mutants by replacing the tyrosine at site 380 either with aspartic acid (Y380D) to mimic permanent phosphorylation or with glycine (Y380A) to mimic permanent dephosphorylation. We then used plasmids to transfect the mutants into HEK293T cells. Comparison of the mutants with WT‐RIPK1 cells revealed decreased binding between SHP2 and RIPK1^Y380A^ but enhanced binding in cells overexpressing RIPK1^Y380D^ (Figure 8G).

Many sites on RIPK1, such as Ser25 and Tyr384, are phosphorylated to inhibit its kinase activity.^[^ ^44^, ^47^ ^]^ Therefore, we also investigated how the dephosphorylation of RIPK1^Tyr380^ would affect RIPK1 kinase activity. Functionally, dephosphorylating RIPK1 at the Tyr380 site enhanced the Ser166 phosphorylation of RIPK1, which increased RIPK1 enzyme activity (Figure 8H). Moreover, SHP2 overexpression promoted RIPK1 activation, as indicated by an increase in the phosphorylation of RIPK1 at Ser166 (Figure 8I). The permanent dephosphorylation of Y380 (Y380A) increased both RIPK1 activity and inflammatory endothelial cell activation, as shown by increases in phosphorylation of RIPK1 at Ser166 and its downstream targets p‐c‐Fos, p‐c‐Jun, ICAM1, IGF1, and CXCL10/13 (Figure 8J,K), whereas the permanent phosphorylation mutant of Y380D had the opposite results. Collectively, all the results supported the idea that SHP2 enhanced RIPK1 enzyme activity by dephosphorylating RIPK1^Tyr380^. The activated RIPK1 then phosphorylated its downstream AP‐1 transcription factor complex. The activated AP‐1 then promoted the transcription of IGF1 and other inflammatory mediators in activated endothelial cells.

### 2.9 Pharmacologic Inhibition of SHP2 Alleviates Sterile Inflammation and Endometrial Hyperplasia in Mice

**Figure 9:**
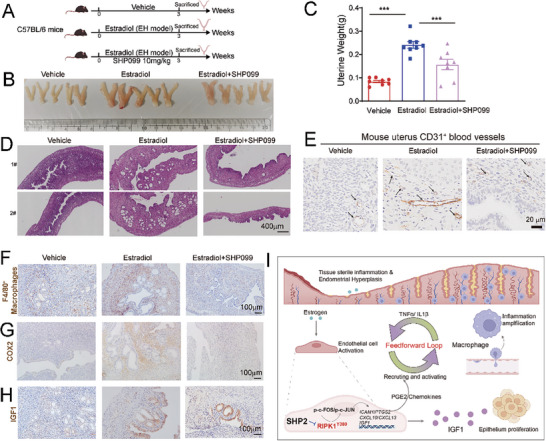
SHP2 inhibition alleviated estradiol‐induced EH in mice. A) Experimental design depicting the 3 groups. B) The morphology of the uteruses of WT mice treated with vehicle (n = 8), estradiol (n = 8, 100 µg kg^−1^), or estradiol plus SHP099 (estradiol, 100 µg kg^−1^; SHP099, 10 mg kg^−1^, n = 8) for 21 days. C) The weights of the mouse uteruses following the indicated treatment. D) H&E staining of the mouse uteruses following the indicated treatment. E) IHC staining of CD31^+^ vessels in mouse uteruses following the indicated treatment. F) IHC staining of F4/80^+^ macrophages in mouse uteruses following the indicated treatment. G) IHC staining of COX2 expression in mouse uteruses following the indicated treatment. H) IHC staining of IGF1 in mouse uteruses following the indicated treatment. I) Schematic illustration of the role of the SHP2‐RIPK1‐AP‐1 axis in tissue sterile inflammation mediated by vascular endothelial cell activation during EH progression. Data are presented as mean ± SEM. Data were analyzed using the Tukey–Kramer test. ^***^ *P* value < 0.001.

We also explored whether pharmacologically targeting SHP2 using its allosteric inhibitor SHP099 could alleviate estradiol‐induced EH in mice. Similar to the results shown in Figure 2, SHP099 treatment significantly reduced the uterine size and weight in EH mice compared to the EH model group (**Figure** **9**A–C). H&E staining showed that SHP099 treatment diminished the architectural abnormalities and reduced the gland‐to‐stroma ratio of mouse uteruses (Figure 9D). The uterine microvascular structure showed less abnormality in the SHP099‐treated mice (Figure 9E). The FACS and IHC results both demonstrated decreased infiltration of macrophages into the uterus after SHP2 inhibition (Figure S10A,B, Supporting Information; Figure 9F). The expression of COX2, an indicator of tissue inflammation, declined after SHP099 treatment, supporting the resolution of endometrial inflammation (Figure 9G). The levels of the IGF1 growth factor also increased in the EH mouse uteruses but decreased in the SHP099‐treated mice (Figure 9H). We also observed an inhibition of the activation of endothelial cells (CD31^+^ICAM1^+^) and an increase in RIPK1 activation in the uteruses of mice given an SHP099 treatment (Figure S10C,D, Supporting Information). All these results supported the possibility that targeting SHP2 could alleviate tissue inflammation and thus EH progression in mice. Taken together, our data verified that SHP2 promotes endothelial cell activation through the dephosphorylation of RIPK1 at the Y380 site, thereby mediating macrophage recruitment and subsequent tissue long‐term sterile inflammation. The inflammatory environment promotes further activation of endothelial cells, causing them to secrete increasing amounts of growth factor IGF1, which promotes epithelial cell proliferation and EH progression in a paracrine manner (Figure 9I).

## 3 Discussion

The response of SHP2 to estradiol has not been reported previously, nor has a role or underlying mechanism been proposed for SHP2 regarding hormone‐induced sterile inflammation in EH. Therefore, the findings presented here are important, as they begin to reveal how estrogen induction of endothelial SHP2 may regulate immune cell responses in the uterus and how crosstalk between endothelial cells, immune cells, and epithelial cells may modulate the as yet largely unexplained progression of EH. More importantly, our study identifies a potential new non‐hormone medical strategy that could replace progestin therapy, which is currently the only medical treatment for EH diseases.

In most cases, treatment with progestogens like progestin is effective in achieving regression of EH without atypia. However, some women do not respond to or are resistant to either oral or local intrauterine progestogen treatments, suggesting that factors other than estrogen may regulate EH diseases. Sterile inflammation of the endometrium environment may be an important risk factor. The link between estrogen signaling and inflammation in endometrial diseases has been previously explored, but few reports have implicated uterine inflammation in the progression of endometrial hyperplasia. Very few reports have considered endometrial hyperplasia as an inflammatory disease, and no study has yet identified the mechanisms driving the underlying molecular pathology of EH. Our study is the first to connect SHP2‐mediated sterile inflammation and the progression of endometrial hyperplasia. Our findings also reveal the potential mechanisms underlying this connection and consequently identify some promising targets for the non‐hormone medical treatment of EH.

Currently, the mechanisms of sterile endometrium inflammation in EH are unknown, making finding a target for regulating inflammation a substantial challenge. Therefore, further understanding of the molecular pathology of tissue sterile inflammation is urgently needed. Here, we have elucidated that endothelial cell activation mediated by SHP2 plays a role in endometrial tissue inflammation and EH diseases. Therefore, targeting endothelial inflammatory activation through SHP2 inhibition, Tyr380 phosphorylation of RIPK1, or even through the use of other anti‐inflammation targets may be promising strategies for EH medical intervention. This idea is supported by a recently published study showing that targeting the tyrosine phosphorylation of endothelial VEGFR was a potential anti‐inflammatory strategy.^[^ ^22^ ^]^

Similarly, a previous study reported that genistein, an inhibitor of protein‐tyrosine kinases, was somewhat effective in EH treatment,^[^ ^48^ ^]^ again indicating that protein tyrosine phosphorylation may be a key regulator in EH.

The abnormal expression, enzyme activity alteration, and constitutive activation or inactivation mutations of SHP2 often occur during sterile inflammation. Many different SHP2 inhibitors have undergone clinical trials for the treatment of cancer, confirming SHP2 as a targetable molecule for medical purposes. However, the functions of SHP2 in epithelial cells, immune cells, fibroblasts, chondrocytes, and endothelial cells vary due to specific disease conditions. In endothelial cells, SHP2 is widely reported to regulate cell apoptosis,^[^ ^49^ ^]^ destabilization of endothelial junctions,^[^ ^50^ ^]^ angiogenesis,^[^ ^51^, ^52^ ^]^ recovery of endothelial adherent junctions,^[^ ^53^ ^]^ and maintenance of the endothelial cell barrier.^[^ ^54^ ^]^ However, the mechanism by which SHP2 regulates endothelial cell activation and the pathway by which endothelial cell activation mediates sterile inflammation after estrogen stimulation are unknown. Here, we show that estradiol increases SHP2 expression in endothelial cells and consequently triggers cell activation through the dephosphorylation of RIPK1 at the Tyr380 site. A previous study reported that dephosphorylation of RIPK1 at the Tyr384 (human, isoform 1) site enhanced the kinase activity.^[^ ^44^ ^]^ Similarly, our results show that the dephosphorylation of RIPK1 (isoform 2) at Tyr380 also promotes the activation of RIPK1, as shown by the increased phosphorylation of Ser166.

Notably, RIPK1 is reported to be a key molecule in type II endothelial activation^[^ ^21^ ^]^ and sterile inflammation.^[^ ^46^, ^55^ ^]^ In our study, the activated form of RIPK1 promotes the activation of transcription factor AP‐1, thereby inducing the transcription of new genes coding for pro‐inflammatory cytokines, chemokines, and *IGF1* in HUVECs. The increases in cytokines and chemokines then reshape the inflammatory environment in the endometrium. However, we have not investigated the precise events that would explain how SHP2 increases after estradiol treatment. We also found that the expression of SHP2 in uterine epithelial cells does not change between healthy individuals and EH patients, while also confirming that estradiol treatment does not increase the in vitro expression of SHP2 in hEECs. These findings indicate that the response of SHP2 to estrogen may differ depending on the cell type and the environment. Interestingly, we unexpectedly found that estradiol alone does not significantly promote the proliferation of hEECs, whereas it does promote the expansion of human endometrial organoids. This surprising result suggests that other factors cooperate with estradiol to promote epithelial cell proliferation. In our study, SHP2 hyperactivation and sterile inflammation in the uterus were driven by extra estrogen but not by gene mutation.

Many studies have reported that the abnormal activation of endothelial cells leads to tissue inflammation and that the association between inflammation and endometrial hyperplasia has been recognized for quite a long time.^[^ ^21^ ^]^ Pro‐inflammatory cytokines have been confirmed to increase in EH tissue,^[^ ^56^ ^]^ and sex steroid hormones are believed to regulate the production and release of cytokines by activating MyD88‐dependent TLR‐ and/or IL‐1‐receptor signaling pathways.^[^ ^57^ ^]^ However, how estrogen induces sterile inflammation and promotes EH remains unclear. Here, in line with previous studies, we also observed significant increases in TNF‐α and IL‐1*β* in the uterus of the EH mouse model. We recognized the important role of immune cells in inflammation, and our analysis identified a significant increase in the interaction between endothelial cells in EH patients. Therefore, we chose to focus on macrophages and further investigate how macrophages affect EH progression.

We found that macrophages are trafficked into the endometrial tissue and activated sequentially by endothelial cells. In turn, the activated macrophages enhance endothelial inflammation in a feedback manner, causing endothelial cells to release more IGF1. The increased level of IGF1 then promotes the over‐proliferation of uterine epithelial cells, but this can be prevented by SHP2 depletion or inhibition, which then reduces inflammation and macrophage infiltration. Nevertheless, other immune cells, such as neutrophils and T cells, are also likely to play roles in EH. Our future studies will be aimed at elucidating the function of other immune cells in the sterile inflammation associated with EH.

Excess estradiol induces simple endometrial hyperplasia, a local hypoestrogenic‐mediated disease. However, as sterile inflammation progresses in complex and atypical EH, inflammation in EH is considered a factor in the promotion and progression of EH pathology, as well as an attributed risk factor for malignancy transformation.^[^ ^18^ ^]^ Non‐steroidal anti‐inflammatory drugs, such as COX2 inhibitors, are reported to reduce the risk of developing certain cancers by regulating cancer‐related inflammation.^[^ ^57^ ^]^ In our study, we validated that the activation of endothelial cells generated and sustained long‐term sterile inflammation to promote EH progression by collecting mouse uteruses at different times and withdrawing estradiol after the construction of the three‐week mouse EH model. Interestingly, the observed EH pathology did not regress after estradiol withdrawal due to the presence of tissue inflammation. We also demonstrated that sterile inflammation was the main risk factor for EH progression at the later stage, which was estradiol independent, as indicated by the similar estradiol levels seen at the later stages of estradiol withdrawal and in the vehicle control mice. Therefore, our study shows that the continuously activated endothelial cells are what sustain the length of the sterile inflammation period, as those cells continuously secrete IGF1 to promote EH progression. Targeting inflammatory mediators, key transcription factors involved in inflammation (such as AP‐1), and/or molecules regulating estrogen‐induced sterile inflammation signaling (SHP2, RIPK1) may decrease the incidence and progression of EH or other estrogen‐related diseases.

## 4 Conclusion

Taken together, our results demonstrate that the high level of SHP2 in endothelial cells promotes cell activation and generates long‐term sterile tissue inflammation by promoting the trafficking of activated tissue macrophages. The macrophages in the inflammatory endometrium further amplify the continuous inflammatory activation of endothelial cells, independent of the further presence or absence of estradiol stimulation. Activated endothelial cells release more IGF1, leading to the abnormal proliferation of endometrial epithelial cells and the progression of EH. Therefore, our study demonstrates a key role for SHP2‐mediated endothelial cell activation in the chronic sterile inflammation associated with EH progression. Targeting SHP2 to control sterile tissue inflammation by regulating endothelial cell inflammatory activation mediated by RIPK1 may represent an effective and promising nonhormonal strategy for treating estrogen‐related diseases.

## 5 Experimental Section

### Mice and the Endometrial Hyperplasia Mouse Model

SHP2^iECKO^ mice were generous gifts from Professor Yuehai Ke (University of Zhejiang, Hangzhou, China). SHP2^iECKO^ mice were generated by crossing SHP2^f/f^ mice with Cdh5‐CreERT2 mice (C57BL/6). SHP2^iECKO^ mice were intraperitoneally injected with tamoxifen (60 mg kg^−1^) for 5 days and left untreated for 7 days to delete endothelial SHP2. SHP2^f/f^ mice were treated with the same dose of tamoxifen as controls. WT C57BL/6 mice were purchased from GemPharmatech Co., Ltd. (Nanjing, China). All mice were given free access to standard laboratory chow and water under a condition of 25 °C, suitable humidity, 12 h dark/light cycle. All animal protocols were carried out according to the NIH Guide for the Care and Use of Laboratory Animals and were approved by the Experimental Animal Care and Use Committee of Nanjing University (IACUC‐2206005). To establish the 17β‐estradiol (estradiol, E2)‐induced endometrial hyperplasia (EH) mouse model, female WT mice aged 6–8 weeks or transgenic mice were subcutaneously injected with 100 µg kg^−1^ estradiol dissolved in olive oil daily for 21 consecutive days. Mice were sacrificed 1 day after the final treatment, and the uteruses were collected for weight determinations and H&E staining. The severity of endometrial hyperplasia was measured by evaluating the uterine size, uterine weight, and any architectural abnormalities.

### Cells

HUVECs were isolated from umbilical cord veins by dissociating with 0.2% (w/v) collagenase II (1 mg mL^−1^) at 37 °C water bath for 20 min. The cells were collected and cultured with Endothelial Cell Medium (ECM; Science ll, Cat.NO. 1001) supplemented with Endothelial Cell Growth Supplement (ECGS). HUVECs between passages 3 and 8 were used in all experiments. Primary lung endothelial cells were isolated from 6‐ to 8‐week‐old mice and from SHP2^f/f^ or SHP2^iECKO^ mice to confirm the efficiency of SHP2 depletion. The lung tissues were cut into pieces and dissociated with a solution containing type‐I collagenase (2 mg mL^−1^, Gibco) and DNase (10 µg mL^−1^, Roche) in Dulbecco's phosphate buffered saline (DPBS) for 45 min at 37 °C. The single‐cell suspension was passed through a 40‐µm cell strainer. The CD31^+^ cells were magnetically separated from other cells using anti‐CD31 microbeads (Miltenyi, Germany, Cat. 130‐097‐418). The CD31^+^ cells were then stained with anti‐mouse CD45 and anti‐mouse CD31 and subjected to FACS sorting to isolate the CD45^−^CD31^+^ endothelial cells. The human endometrial epithelial cell line (hEEC, WHELAB C1225) was purchased from Shanghai Whelab Bioscience Limited and cultured with MEM supplemented with 10% fetal bovine serum (FBS) and 1% nonessential amino acids.^[^ ^43^ ^]^ HEK293T cells and THP1 cells were purchased from ATGG and cultured with DMEM and RPMI1640 medium separately supplemented with 10% FBS and 1% penicillin/streptomycin.

### Clinical Samples

The human endometrium tissues were obtained from EH patients. Detailed information about the EH patients is listed in Supplementary Table S1. The study was approved by the Ethics Committee of Tongde Hospital of Zhejiang Province (No. 2021013).

### Organoid Culturing from Endometrial Tissue

The human endometrial organoids following published protocols were generated.^[^ ^36^, ^37^, ^58^ ^]^ Briefly, endometrial tissues were obtained from patients with EH after informed written consent. On receipt, the tissues were minced into 0.5–1 mm^3^ pieces and extensively rinsed in Ca^2+^/Mg^2+^‐free PBS. The tissue samples were then dissociated with collagenase IV (2 mg mL^−1^) solution containing a ROCK inhibitor (RI; Y‐27632, 10 µm) for 1 h at 37 °C in a rotatory shaker. Subsequently, the tissue was digested in TrypLE supplemented with RI for another 15 min. After digestion, the enzymes were stopped by medium dilution. The cell suspension was filtered through a 70 µm cell strainer and centrifuged at 500 g for 5 min to obtain the cell pellet. The cell pellet was resuspended in 70% Matrigel/30% endometrial organoid medium (356231, Corning). The compounds in the endometrial organoid medium are listed in Table S4 (Supporting Information). ≈20–25 µL drops of Matrigel/cell suspension were dropped into the center of the wells of a 96‐well plate and inverted for 30 min. The endometrial organoid medium was added to the wells and was replaced every 3 days. The organoids were passaged every 7–10 days based on their size. The treatment on organoids was performed on day 3 after seeding them in the 96‐well plate for the indicated time in each experiment. Organoids of low passage number (P1‐P4) were used for all the experiments described.

### Single Cell Dissociation, scRNA‐Seq Library Construction, and Sequencing for Endometrium Samples

Human endometrial tissues were digested and isolated as described previously.^[^ ^43^ ^]^ Dissociated single cells were stained with acridine orange/propidium iodide (AO/PI) for viability assessment using a Countstar Fluorescence Cell Analyzer. The proportion of living cells was greater than 85%. The scRNA‐Seq libraries were generated using the 10× Genomics Chromium Controller Instrument and Chromium Single Cell 3′ V3.1 Reagent Kits. All the libraries were sequenced using an Illumina sequencer (Illumina, San Diego, CA, USA).

### Single‐Cell Sequencing Data Processing

The 10× Chromium single‐cell RNA sequencing (scRNA‐seq) data in this study were processed using CellRanger (v3.1.0; 10× Genomics) for read alignment, barcode assignment, and unique molecular identifier (UMI) counting, based on the corresponding reference genome (GRCh38). Filtered count matrices from the CellRanger pipeline were converted to sparse matrices using the Seurat package (v4.0.0). Cells were filtered with a UMI count greater than 200 and a mitochondrial percentage less than 20%. The “doubletFinder_v3” method from the DoubletFinder package (v2.0.3) was applied for additional cell filtering. Filtered data were then log normalized and scaled, with cell–cell variation due to UMI counts and percent mitochondrial reads regressed out. Batch effects among samples and experiments were avoided using Seurat's canonical correlation analysis (CCA) integration tool to integrate single‐cell data. The top 2000 most variably expressed genes were determined using the “vst” method in the “FindVariableFeatures” function and scaled using “ScaleData” with regression on the proportion of mitochondrial UMIs (mt.percent). Cell clustering was performed using the “FindClusters” function at a resolution of 1.5. Dimensionality reduction was performed with the “RunUMAP” function and visualized with Uniform Manifold Approximation and Projection (UMAP). For subgroup cell clustering, cells of different types were extracted separately and clustered by their respective top 20 principal components (PCs) using different resolutions based on visual inspection. Marker genes for each cluster were identified using the Wilcoxon rank‐sum test (“FindAllMarkers” function with default parameters) and each cell cluster was annotated based on known marker genes from the top gene list.

### Pathway Analysis

Differentially expressed genes (DEGs) were detected by the “FindAllMarkers” function in Seurat. All the gene‐set enrichment analyses (GSEA) on DEGs in this study were performed using the clusterProfiler (v3.14.3) package.

### Cell–Cell Interaction Analysis

Cell–cell interactions among the cell types were estimated using CellPhoneDB (v2.1.7) with default parameters (20% of cells expressing the ligand/receptor) and using version 2.0.0 of the database. CellPhoneDB infers the potential interaction strength between two cell subsets based on the gene expression level of a receptor–receptor pair. The significance of the interaction is assessed through a permutation test (1000 times) using the normalized gene expression as input. Interactions with a *p*‐value < 0.05 were considered significant. Only ligand–receptor interactions based on the annotation from the CellPhoneDB database were considered, and receptor–receptor and other interactions without a clear receptor was discarded.

### Gene‐Regulatory Network

To identify cell‐type and organ‐specific gene regulatory networks, a Single‐cell Regulatory Network Inference and Clustering (v0.11.2; a Python implementation of SCENIC) using the dataset were performed. The original expression data were first normalized by dividing the gene count for each cell by the total number of cells in that cell and multiplying by 10 000, followed by a log1p transformation. Next, the normalized counts were used to generate the co‐expression module with the GRNboost2 algorithm implemented in the arboreto package (v0.1.6). Finally, pySCENIC was used with its default parameters to infer co‐expression modules using the above‐created RcisTarget database. An AUCell value matrix was generated to represent the activity of the regulators in each cell. Gene regulatory networks (GRNs) were visualized using the igraph package in R.

### Immunoblotting, Immunofluorescence Staining, and Coimmunoprecipitation

For immunoblotting, cells were collected and lysed with WB and IP lysis buffer (Beyotime Biotechnology, China). The extracted proteins were quantified using bicinchoninic acid (BCA). Equal quantities of proteins from different samples were separated on SDS‐PAGE gels and transferred onto polyvinylidene difluoride membranes. The cropped membranes were hybridized with different primary antibodies at 4 °C overnight, washed three times with phosphate‐buffered saline containing 0.1% Tween20 (PBST), and incubated with the secondary antibodies of the corresponding species for 1 h. After three washes, the bands were immunoblotted using an enhanced luminescence (ECL) kit. The primary antibodies used are listed in Table S2 (Supporting Information).

The tissue sections were deparaffinized and rehydrated, and the antibodies were retrieved with sodium citrate. For cell immunofluorescence, the cells were fixed with 4% paraformaldehyde (PFA) and permeabilized with 0.5% Triton‐X100 for 15 min. The samples were blocked with 5% goat serum in PBST and incubated overnight at 4 °C with primary antibodies. After three washes, the samples were incubated for 1 h at room temperature with the secondary antibodies (Table S2, Supporting Information). For immunohistochemistry, proteins were detected using the Real Envision Detection kit (GeneTech) according to the manufacturer's instructions. The nuclei were stained with hematoxylin. For immunofluorescent staining, the nuclei were stained with 4′,6‐diamidino‐2‐ phenylindole (DAPI) (Beyotime). The samples were then mounted using Fluoromount‐G (Southern Biotech), and images were acquired using a confocal microscope. The images were further processed using Image J software.

### For Coimmunoprecipitation

Equal amounts (1 mg) of proteins from each sample were incubated with 2 µg of primary antibody at 4 °C overnight and then precipitated with magnetic Protein A/G beads (Millipore) at 4 °C for 4 h. Proteins not binding to the beads were washed away by six washes with cold PBS and two washes with cold lysis buffer. The beads were then boiled for 10 min in SDS loading buffer. The targeted protein was detected by western blotting with the indicated antibodies (Table S2, Supporting Information).

### SHP2 Enzyme Activity Assay

HUVECs were treated with different doses of estradiol for 48 h, lysed in WB and IP lysis buffer supplemented with phenylmethylsulfonyl fluoride (PMSF) and a protein phosphatase inhibitor (Roche), and quantified using the BCA assay. Proteins (2 µg) were dissolved in a fixed volume of 100 µL with buffer (60 mM HEPES, pH 7.2, 75 mM NaCl, 75 mM KCl, 1 mM EDTA, 0.05% P‐20, and 5 mM DTT) in a 96‐well black plate at 25 °C for 30 min, and the surrogate 6,8‐difluoro‐4‐methylumbelliferyl phosphate (DiFMUP) substrate (Invitrogen) was added and incubated at 25 °C for another 30 min. The fluorescence signal was measured using a microplate reader (Envision) with excitation and emission wavelengths of 340 and 450 nm, respectively. A group with proteins and PHPS1 (20 µM, a SHP2 inhibitor) as a control to exclude the effects of all other protein phosphatases was also set up. The difference between these two groups represented the enzyme activity of SHP2 in HUVECs after estradiol treatment.

### Quantitative PCR

Total RNA was extracted from cells or uteruses using Trizol reagent following the manufacturer's procedure. Reverse transcription to cDNA was performed using 1000 ng of RNA and Hiscript II qRT SuperMix for qPCR (Cat#: R223, Vazyme Biotech Co., Ltd., China). Quantitative RT‐PCR was conducted using Taq Pro Universal SYBR qPCR Master Mix (Cat#: Q712, Vazyme Biotech Co., Ltd., China) on a CFX 100 cycler (Bio‐Rad, Hercules, CA). The primers are listed in Table S3 (Supporting Information). Gene expression was calculated using the equation RQ = 2^−△△Ct^ and normalized to *GAPDH* or *ACTB*.

### ELISA Assays

Cell culture supernatant was collected and used to detect IGF1 levels using a Human IGF‐1 ELISA Kit according to the manufacturer's procedure (Multisciences, EK1131). Mice sera were collected and diluted 2 or 5 times for detection of estradiol levels at different times using a QuicKey Pro Mouse E2 (Estradiol) ELISA Kit according to the manufacturer's procedure (Elabscience, E‐OSEL‐0008).

### Monocyte Adhesion Assay

THP1 cells were first labeled with CM‐Dil (2.5 µg mL^−1^) for 10 min at a density of 1 × 10^6^ cells per mL and then 1 mL of the labeled THP1 cells was incubated with HUVECs with different treatments for 60 min at 37 °C. Nonadherent THP1 cells were washed away gently three times with PBS. Fluorescence images of adherent THP‐1 cells were captured and analyzed using ImageJ software to calculate the number of adherent monocytes.

### CCK8 and EdU Incorporation Assay

For EdU incorporation assays, hEECs were seeded in a 24‐well plate at a density of 4 × 10^4^ per well and cultured overnight, followed by the indicated treatment for the indicated time. 5‐Ethynyl‐2′‐deoxyuridine (EdU; Beyotime, 20 µM) was added into the medium for 3 h at 37 °C and the cells were then fixed in 4% PFA and permeabilized with 0.2% Triton‐X100. After three washes with PBS, the cells were incubated (while protected from light) using a click additive solution and nuclei were stained with DAPI. Images were obtained using a fluorescence microscope.

### Cytoplasmic Protein and Nuclear Protein Extraction

HUVECs were cultured in 10 cm dishes and treated with estradiol or dimethylsulfoxide (DMSO) for 48 h. The cells were then released using trypsin and washed with PBS. The cells were lysed by adding 400 µL of 1% NP40 buffer (10 mM Tris‐HCl, pH = 7.5) and then the cell lysis was added to 1 mL of 24% sucrose solution drop by drop and centrifuged at 2500 g for 10 min. The supernatant containing cytoplasmic proteins was collected and the uppermost 200 µL of cytoplasmic proteins were used. The precipitate was washed with EDTA‐PBS solution (0.5 mM) at 4 °C by centrifuging at 3500 g for 10 s and then washed with PBS for 30 s. The remaining precipitate, consisting of cell nuclei, was lysed with RIPA buffer on ice for 30 min and then centrifuged to obtain a nuclear protein extract.

### RNA‐Sequence Analysis

Total RNA was extracted using Trizol reagent following the manufacturer's procedure. The total RNA quantity and purity were analysis of Bioanalyzer 2100 and RNA 6000 Nano LabChip Kit (Agilent, CA, USA, 5067‐1511), high‐quality RNA samples with RIN number > 7.0 were used to construct the sequencing library. The purified and cleaved RNA fragments were reverse‐transcribed to create the cDNA using SuperScript II Reverse Transcriptase (Invitrogen, cat. 1896649, USA). The resulting cDNA libraries were subjected to 2 × 150 bp paired‐end sequencing (PE150) on an Illumina Novaseq 6000 following the vendor's recommended protocol. RNA‐seq data analysis was performed by LC‐Bio Technology CO., Ltd., Hangzhou, China, using the Cloud Analysis Platform (https://www.omicstudio.cn/).

### SILAC and Mass Spectrometry Analysis

HUVECs were cultured in Lys/Arg free DMEM:F12 medium (Thermo Scientific Cat: 88370) re‐supplemented with heavy, middle, or light Lys and Arg for a minimum of six population doublings. The cells were then incubated with dimethylsulfoxide (DMSO), estradiol (E2), or a combination of E2 and SHP099 for 6 h. After washing the cells three times with cold PBS, 10 cell volumes of 8 m urea lysis buffer (8 m urea, 100 mM NH_4_HCO_3_, protease inhibitors and phosphatase inhibitors (Roche) were added. The cells were incubated for 30 min on ice and then sonicated for 2 s at 5 s interval for 3 min at 30% energy. The sonicated samples were centrifuged at 4 °C by centrifuging at 12 000 rpm for 10 min. The extracted proteins were then digested with trypsin, mixed in equal proportions, and analyzed by mass spectrometry. Post analysis was done using MaxQuant software.

### Statistics

Graphpad Prism 8 software was used for all statistical analyses. Data was assessed for normal distribution and similar variance between groups. The unpaired two‐tailed Student's *t*‐test, Dunnett's *t*‐test, Tukey–Kramer, and Dunnett tests were used to assess statistical significance between two groups. One‐way analysis of variance (ANOVA) with Tukey's multiple comparisons was used when comparing three or more groups. A *P* value less than 0.05 was considered statistically significant. All data are presented as the mean±SEM.

### Ethics Approval and Consent to Participate

All animal experiments in the present study were undertaken in accordance with the National Institutes of Health Guide for the Care and Use of Laboratory Animals, with the approval of the Animal Care and Use Committee of Nanjing University at IACUC‐2206005.

## Conflict of Interest

The authors declare no conflict of interest.

## Author Contributions

J.P., J.Q., and W.F. contributed equally to the work. Y.S., Q.D., and W.L. were responsible for conceptualization. The methodology was carried out by J.P., J.Q., W.F., L.Z., W.Z., L.Z., M.T., and Q. X. J. P. handled the investigation, visualization, and original draft writing, while J.P. and Y.S. contributed to the review and editing of the manuscript.

## Supporting information



Supporting Information

Supporting Information

Supporting Information

Supplemental Movie 1

## Data Availability

The data that support the findings of this study are available in the supplementary material of this article.
