# Supplementary material for: SHP2‐Triggered Endothelial Cell Activation Fuels Estradiol‐Independent Endometrial Sterile Inflammation
Source: Adv Sci (Weinh). 2024 Sep 5;11(41):2403038. doi: 10.1002/advs.202403038 (PMC11538683; doi:10.1002/advs.202403038)
Supplement: Supplementary file 1 — Supporting Information [file ADVS-11-2403038-s001.docx]

Supporting Information

**SHP2-triggered endothelial cell activation fuels estradiol-independent endometrial sterile inflammation**

*Jie Pan^#^, Jiao Qu ^#^, Wen Fang^#^, Lixin Zhao, Wei Zheng, Linhui Zhai, Minjia Tan, Qiang Xu, Qianming Du^*^, Wen Lv^*^, Yang Sun^*^*

**This file includes:**

Supplementary Experimental Methods

Supplementary Figure S1-S10

Supplementary Tables S1-S4

**Experimental Methods**

*Flow cytometry analysis***:** For tissue preparation, mice uteruses were cut into pieces and digestion with solution containing collagenase Ⅳ (2 mg/mL) and DNase I (10 U/mL) at 37 ℃, 180 rpm in a rotatory shaker. Then the cell suspension was filtered using 40-μm cell strainer to get the single cell suspension. For adherent cell, they were digested to single cell by trypsin and washed with PBS. Then cells were performed FCR blocking. Next, for surface marker staining, all antibodies (supplementary Table 2) were used for staining at 4 ℃ for 30 min in the dark. For cell cycle detection, the cells were fixed with 70% cold ethanol for 4 h, then removed RNA with RNase and stained with PI at 4 ℃ for 30 min in the dark. Then cells were washed with FACS buffer 3 times followed by flow cytometry data acquisition (Thermo fisher Attune NXT) and analysis (Flowjo.V10).

*Genotype identification for transgenic mouse:* Genotype identification was performed with Quick Genotyping Assay Kit for Mouse Tail as the instruction (Beyotime, China). Briefly, cut 0.2-1cm mouse tail tip and place it in 100 μL of DNA extraction solution. Placed samples at 55℃ in a water bath or PCR machine and incubated for 15 minutes. Then, incubated for another 5 minutes at 95℃ in a water bath or PCR machine. Added 100 mL Stop Solution to each sample, and mixed well by vortex. Then proceeded to PCR with the indicated primers. Finally, analyzed PCR products by agarose gel electrophoresis. Shp2 mut: 400 bp, Shp2 WT :292 bp. Cdh5-ERT2Cre: 762 bp.

*Intracellular Ca^2+^ measurement:* Intracellular Ca^2+^ was measured by loading Fluo-4 AM into cells and detected on a Zeiss confocal microscope, microplate reader and flow cytometry. Ca^2+^ level in endothelial cells was collected using manually selected regions of interests (ROIs) along the microchannels or on the microplate reader at different time points. Each ROI covered the area of one individual endothelial cell, as indicated by the fluorescence outline. The changes in intracellular Ca^2+^ were quantified by calculating the mean fluorescence intensity of each ROIs or the plate layout after the subtraction of the background autofluorescence.

*For measurement of intracellular Ca^2+^ release and store-operated Ca^2+^ influx^[1]^:* To exclude the extracellular Ca^2+^ in the medium, the HUVECs loaded with Flu-4 AM were supplemented with EGTA (2 mM) before placed on the imaging work station or flow cytometer. We first collected the basal Ca^2+^ signaling in the cytoplasm and then E2 (40 ng/ml) was added into the cell culture dish or flow tube quickly. Thereafter, the Ca^2+^ release from intracellular Ca^2+^ store, maybe ER or mitochondrial, was detected by the microscope or flow cytometer at different time points. The fluorescence declined with time and 9 minutes after E2 stimulation, CaCl_2_ (2 mM) was added into the medium or flow tube quickly. The store-operated Ca^2+^ influx signaling was detected at different time points.

*Differentially expressed genes (DEGs) Analysis for the data of bulk RNA-Seq:* Genes differential expression analysis was performed by DESeq2 software between two different groups. The genes with the parameter of false discovery rate (FDR) below 0.05 and absolute fold change ≥ 2 were considered differentially expressed genes.

*KEGG enrichment analysis for the data of bulk RNA-Seq:* Differentially expressed genes were then subjected to enrichment analysis of KEGG pathways in KEGG database (https://www.kegg.jp/kegg/). Pathway enrichment analysis identified significantly enriched metabolic pathways or signal transduction pathways in DEGs comparing to the whole genome background.

[1] Liu, Y., Y. Ma, J. Xu, G. Zhang, X. Zhao, Z. He, L. Wang, N. Yin, and M. Peng, *VMP1 prevents Ca2+ overload in endoplasmic reticulum and maintains naive T cell survival.* J Exp Med, **2023**. 220(6).


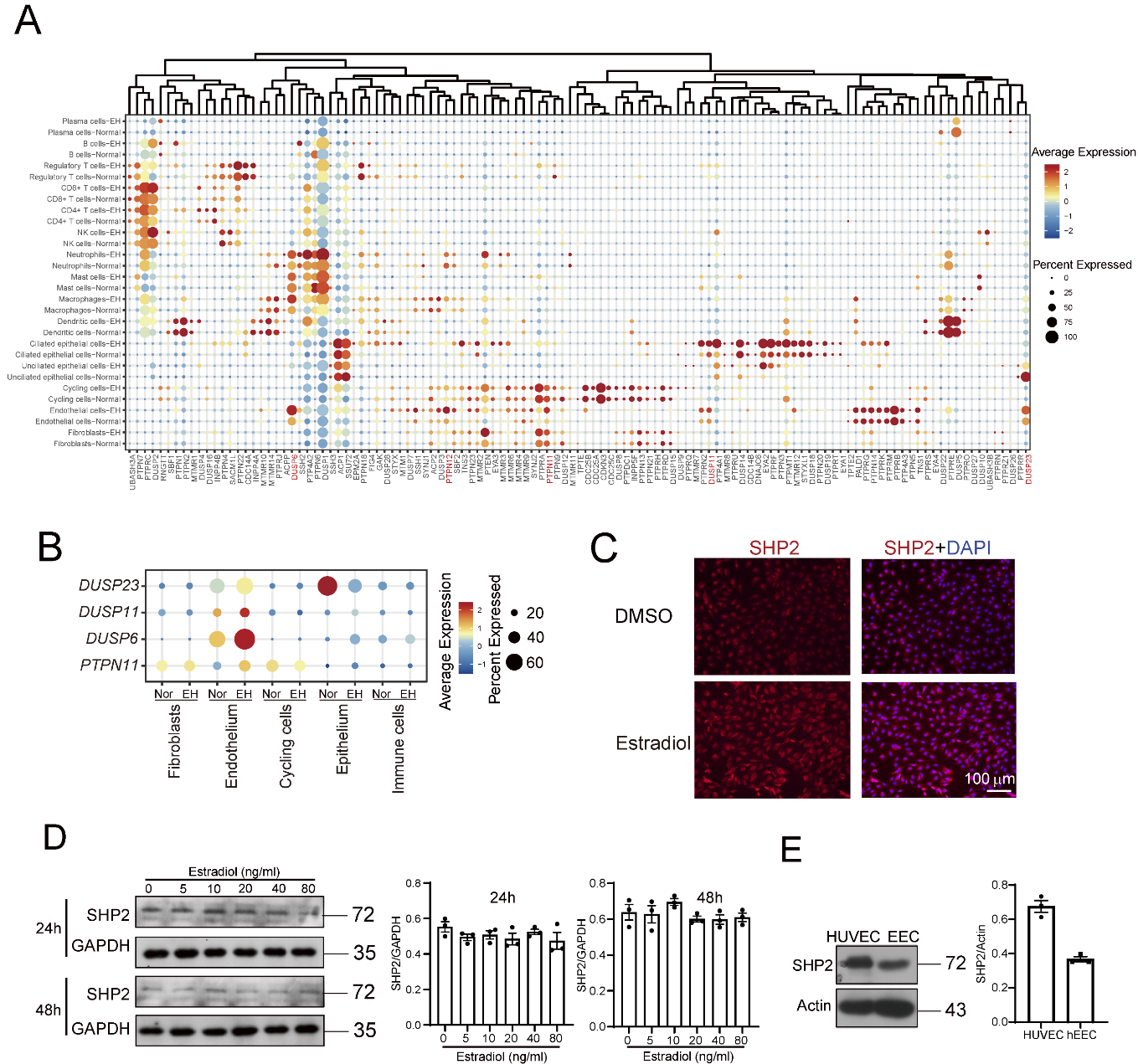


Figure S1. SHP2 expression in endothelial cells in different conditions.

**A** Dot plot showing the different expression pattern of all the 125 protein tyrosine phosphatases in normal and EH samples of all cell types.

**B** Dot plot showing the expression of 4 protein tyrosine phosphatases in different cell types in human endometrium tissue from the scRNA-Seq data.

**C** Immunofluorescence image of SHP2 after estradiol treatment.

**D** Western blot analysis of SHP2 protein expression in hEECs after different dose of estradiol for 24 h or 48 h. n=3. The protein expression of SHP2 was quantified.

**E** The base expression of SHP2 in HUVECs and hEECs. n=3. The protein expression of SHP2 in the two types of cells was quantified.


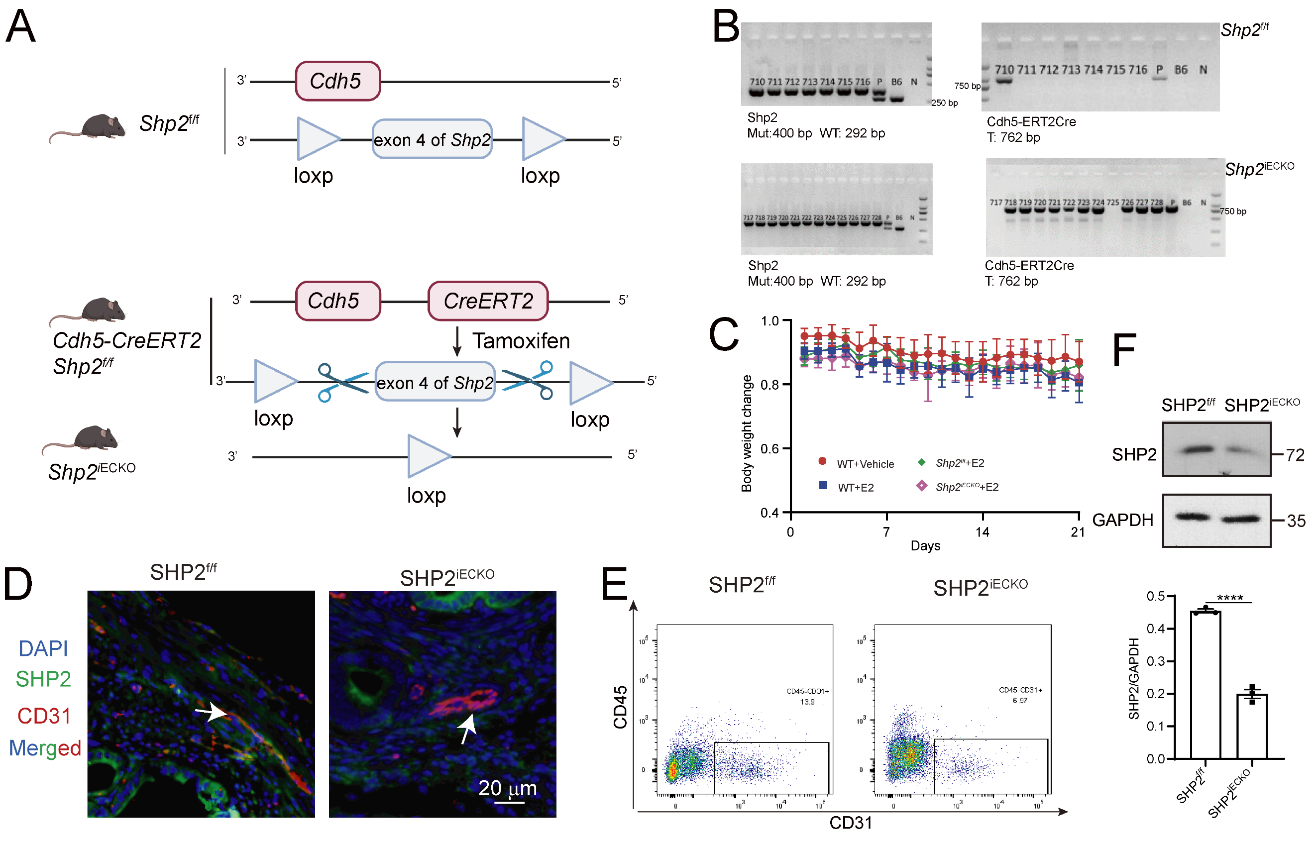


Figure S2. Endothelial specific Shp2 depletion in SHP2^iECKO^ mice.

**A** Construction of tamoxifen-induced endothelial cell specific depletion of Shp2 in C57BL/6 mice, cre-mediated deletion of exon 4 of Shp2 leaded to frameshift mutations.

**B** Genotyping identification of mice were detected by PCR.

**C** Body weight of mice from 4 groups (n=6/group). WT+ Vehicle group represents the WT C57BL/6 mice treated with olive oil for 21 days. WT+E2 group represents the WT C57BL/6 mice treated with estradiol (100 ug/kg) for 21 days. SHP2^f/f^ +E2 group represents the SHP2^f/f^ transgenic mice with C57BL/6 background treated with estradiol (100 ug/kg) for 21 days. SHP2^iECKO^ +E2 group represents the specific endothelial cell SHP2 deletion transgenic mice with C57BL/6 background treated with estradiol (100 ug/kg) for 21 days.

**D** Immunoflurorescence staining of SHP2 (green) in endothelial cell (CD31^+^, red) after tamoxifen injection in mice endometrium. n=6.

**E** FACS sorting of CD45^-^CD31^+^ lung endothelial cells.

**F** WB analysis of SHP2 depletion in CD45^-^CD31^+^ endothelial cells from mice lung after tamoxifen injection. n=3. The protein expression of SHP2 in primary endothelial cells was quantified and statistically analyzed. Student’s *t* test. *****P* value < 0.0001.


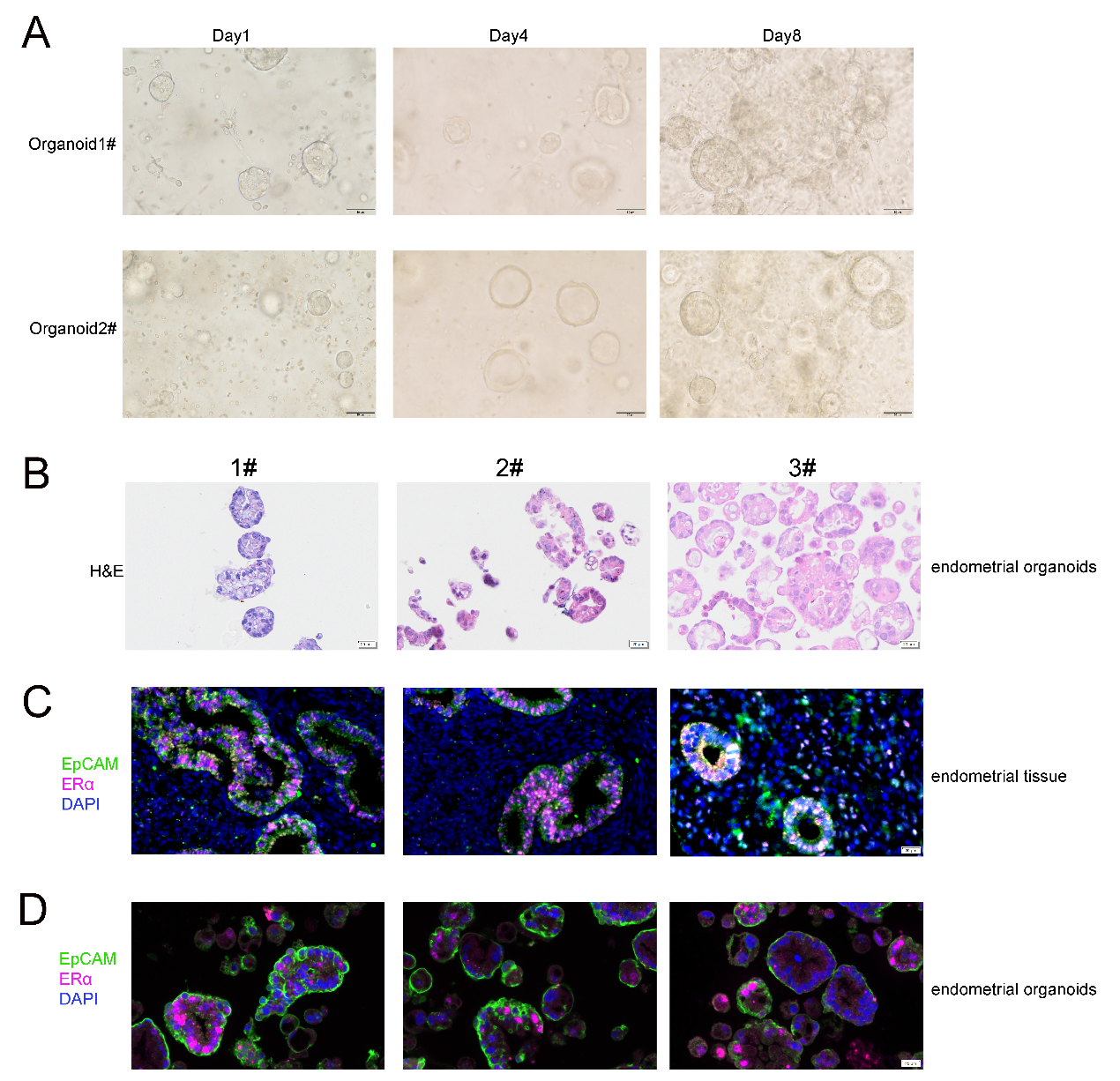


Figure S3. Validation of endometrial organoid on the levels of characteristic protein, structure, and gene.

**A** The morphology of endometrial organoids after seeding in the plate from its primary tissue. N=4. Scale bar: 50 μm.

**B** H&E staining of endometrial organoids. N=3. Scale bar: 20 μm.

**C** Immunofluorescence staining of the expression of ERα (magenta) and EpCAM (green) on the endometrial tissue. N=3. Scale bar: 20 μm.

**D** Immunofluorescence staining of the expression of ERα (magenta) and EpCAM (green) on the constructed endometrial organoids. N=3. Scale bar: 20 μm.


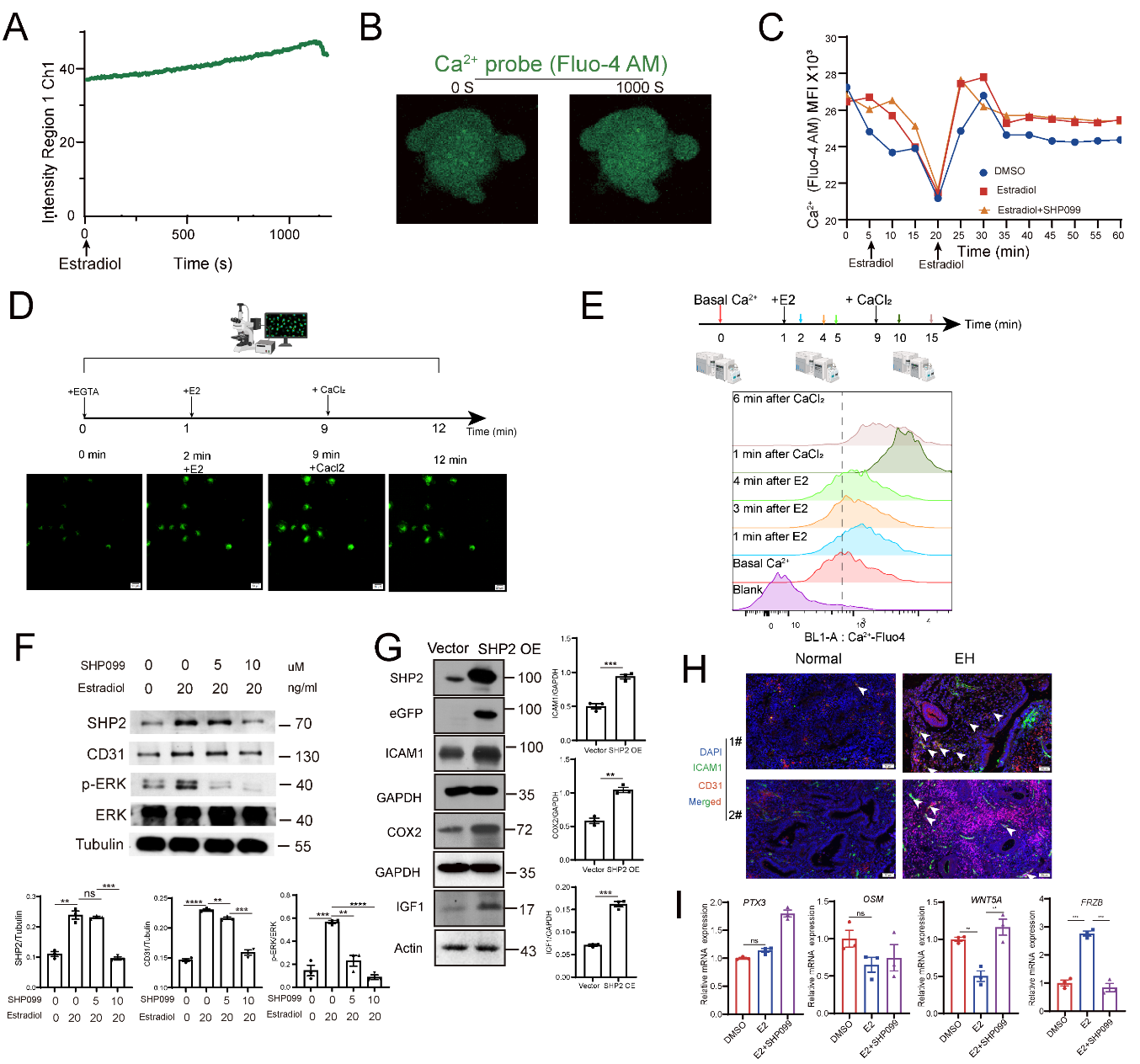


Figure S4. Estradiol promoted endothelial cell activation partly through increased SHP2.

**A** Cytosolic Ca^2+^ levels, as indicated by the fluorescent probe Fluo-4 AM. HUVECs were exposed to E2 (40 ng/mL) for the indicated time.

**B** The representative image of intracellular Ca^2+^ from (A) indicated by green fluorescent probe Fluo-4 AM.

**C** MFI of cytosolic Ca^2+^ levels detected by microplate reader, as indicated by the fluorescent probe Fluo-4 AM.

**D** The intracellular Ca^2+^ release from the most important intracellular Ca^2+^ store, ER, and the extracellular Ca^2+^ influx after supplementing CaCl_2_ (2 mM) into the medium, as indicated by the fluorescent probe Fluo-4 AM. E2 (40 ng/mL).

**E** The intracellular Ca^2+^ release from ER and Ca^2+^ influx after supplementing CaCl_2_ was detected by flow cytometry at different time point. E2 (40 ng/mL), CaCl_2_ (2 mM).

**F** The efficiency of SHP099 (5 or 10 μM) on the inhibition of SHP2 and activation of downstream ERK. The protein expression was quantified. Dunnett’s *t* test.

**G** WB analysis of endothelial cells activation related proteins after SHP2 overexpression in HUVECs. The protein expression was quantified. Student’s *t* test.

**H** Immunofluorescence staining of activated endothelial cell (CD31^+^ICAM1^+^) expression in human normal or EH endometrium tissues.

**I** The effects of estradiol on the mRNA expression of the indicated extracellular proteins in HUVECs. HUVECs were treated with estradiol (E2, 40 ng/mL) or E2 (40 ng/mL) plus SHP099 (5 μM) for 24 h. Data were analyzed by Tukey-Kramer Figure S4I.

**P* value < 0.05; ***P* value < 0.01; ****P* value < 0.001; *****P* value < 0.0001; ns represents no significant difference.


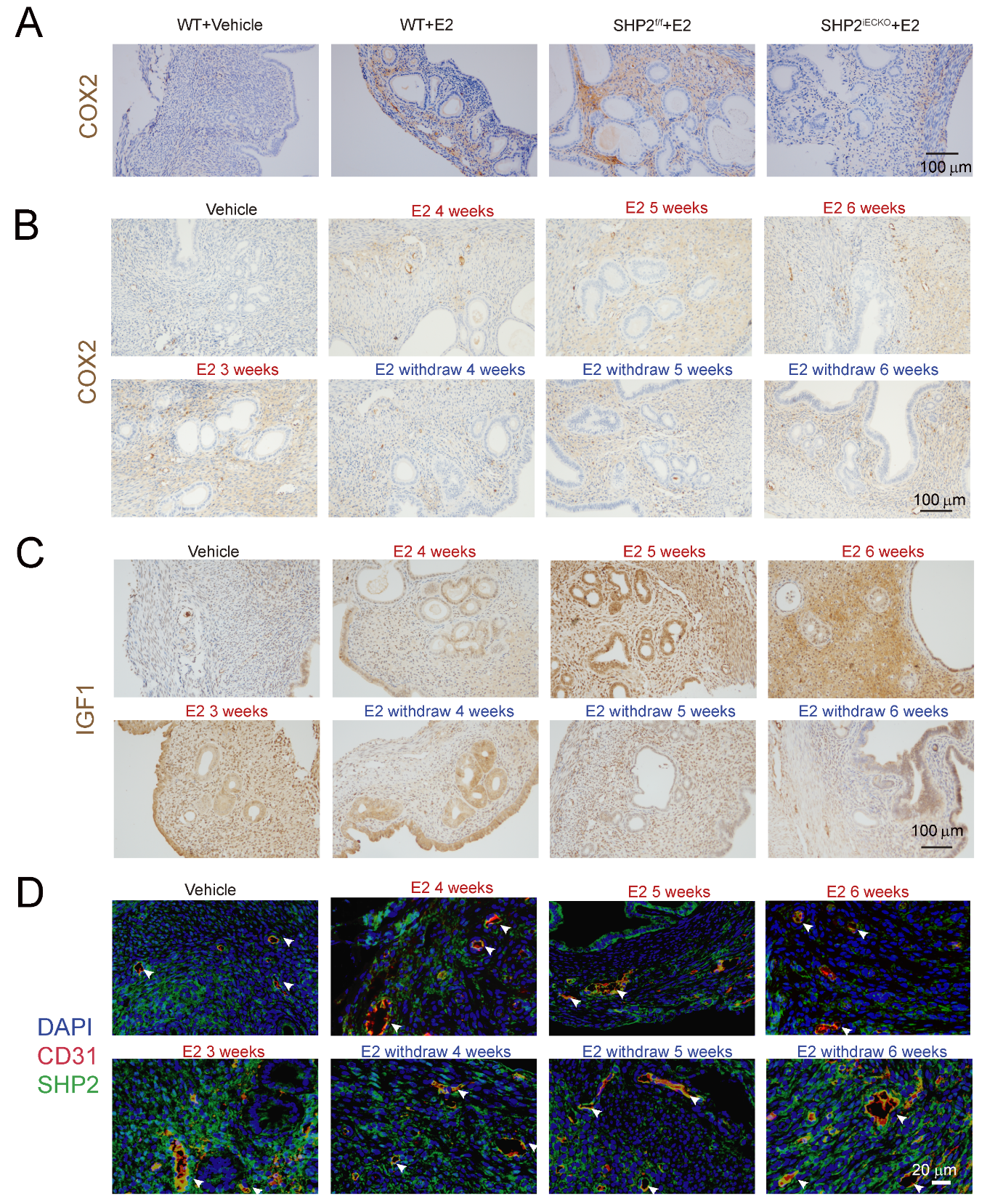


Figure S5. Continuous SHP2 increasement sustained endometrium inflammation and IGF1 secretion.

**A** IHC staining of COX2 expression in the mice uteruses from the 4 indicated groups. n=6/group.

**B-C** IHC staining of the expression of IGF1 and COX2 in mice uterus from indicated groups of different time points. n=6/group.

**D** Immunofluorescence staining of the expression of SHP2(green) in endothelial cells (CD31^+^, red) in the mice uteruses from the indicated group of different time points. n=6/group.


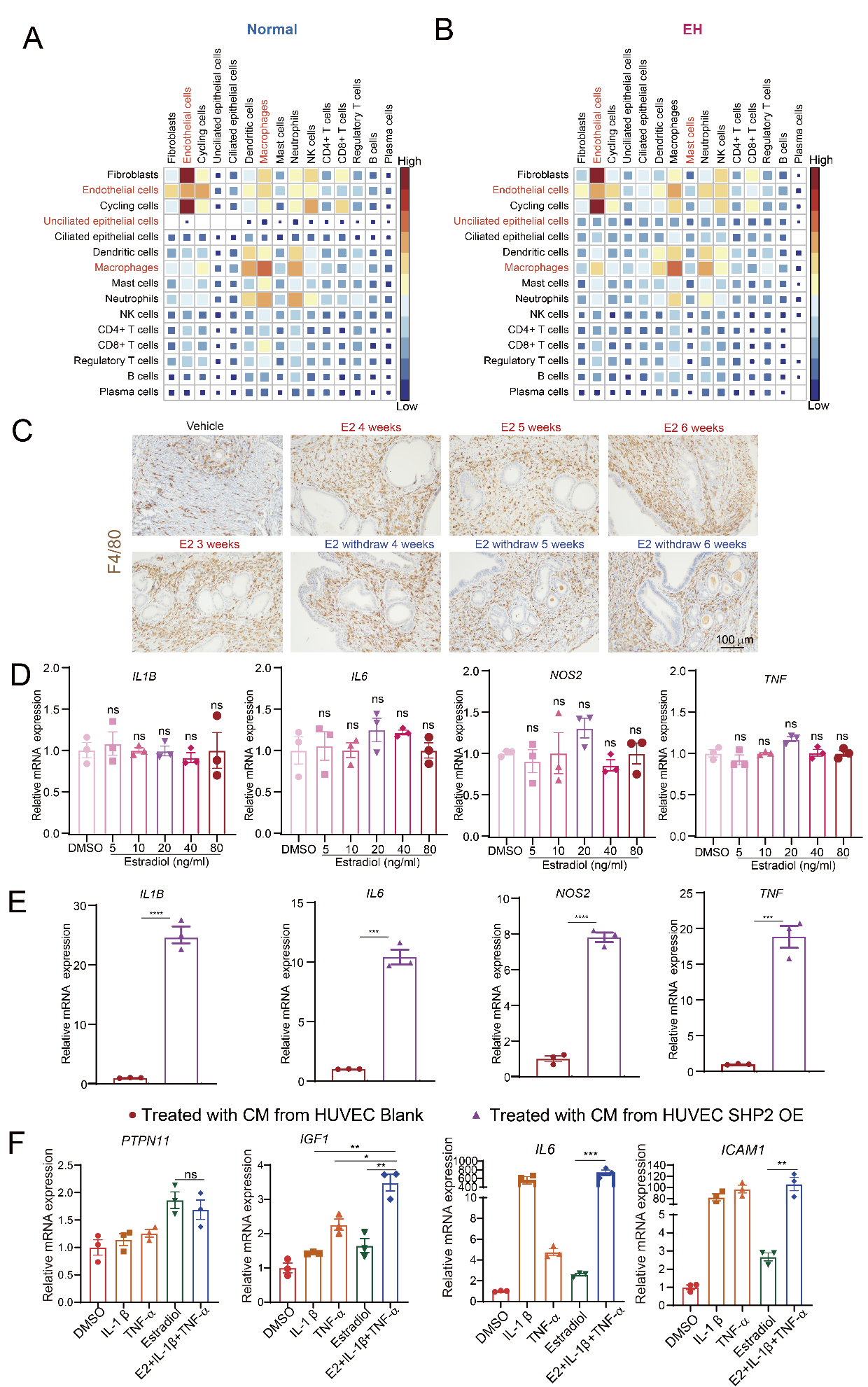


Figure S6. Endothelial SHP2 enhanced endothelial cell-macrophage interaction and promoted macrophage activation.

**A** Heatmap of the cell-cell interactions predicted by CellPhoneDB between different cell clusters in endometrial samples from normal healthy individuals.

**B** The cell-cell interactions predicted by CellPhoneDB between different cell clusters in endometrial samples from EH patients.

**C** IHC staining of the expression of F4/80^+^ in mice uterus from indicated groups of different time points. n=6/group.

**D** The mRNA expression of macrophage activation related genes in PMA activated THP1 cells treated by estradiol. THP1 cells were treated with PMA for 24 h and then subjected to different doses of estradiol for another 24 h. The mRNA expression of indicated genes were detected by qPCR (n=3). All data are shown as the mean ± SEM. ns = no significant difference.

**E** The mRNA expression of macrophage activation related genes in PMA activated THP1 cells cultured with HUVEC condition medium. THP1 cells were treated with PMA for 24 h and then cultured with conditional medium from HUVECs with or without SHP2 overexpression for another 24 h. CM represents condition medium. The mRNA expression of indicated genes were detected by qPCR (n=3).

**F** Gene expression of *PTPN11*, *IL6*, *ICAM1* and *IGF1*gene expression in HUVECs after cytokines treatment (IL-1β, 5 ng/mL; TNF-α, 20 ng/mL) with or without estradiol (E2, 40 ng/mL) for 24 h. All data are shown as the mean ± SEM. Data were analyzed by Dunnett’s *t* test in Figure. S6D, Student’s *t* test in Figure S6E and Tukey-Kramer Figure 6F. **P* value <0.05; ** *P* value < 0.01 ****P* value < 0.001; *****P* value <0.0001; ns represents no significant difference.


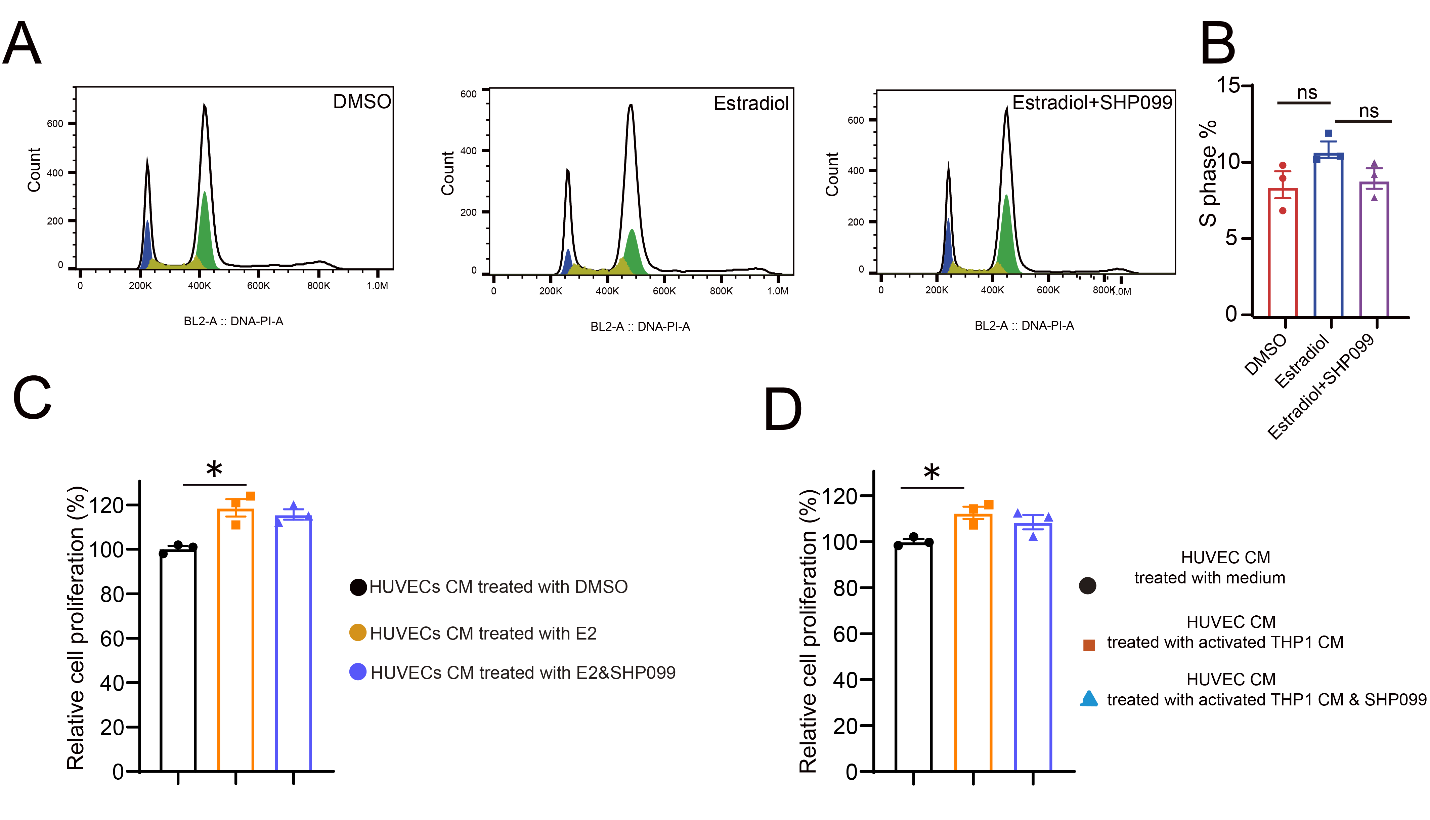


**Figure. S7 The effects of estradiol or HUVEC conditional medium on the proliferation of hEECs.**

**A** Cell cycle distribution of hEECs after indicated treatment for 24 h. hEECs were treated with dimethylsulfoxide (DMSO), estradiol (40 ng/mL) or estradiol (40 ng/mL) plus SHP099 (5 μM) for 24 h and the cells were collected for cell cycle detection.

**B** Statistical analysis of the percentage of S phase of hEECs after indicated treatment.

**C** Detection of cell proliferation by CCK8 assay. hEECs were cultured with conditional medium from HUVECs that were pretreated with estradiol (40 ng/mL), estradiol (40 ng/mL) plus SHP099 (5 μM) for 48 h.

**D** Detection of cell proliferation by CCK8 assay. hEECs were cultured with conditional medium from HUVECs with or without SHP099 that were pretreated with conditional medium from PMA activated THP1, or conditional medium from SHP099 treated THP1 for 48 h. All data were shown as the mean ± SEM. Data were analyzed by Tukey test. **P* value < 0.05; ns represents no significant difference.


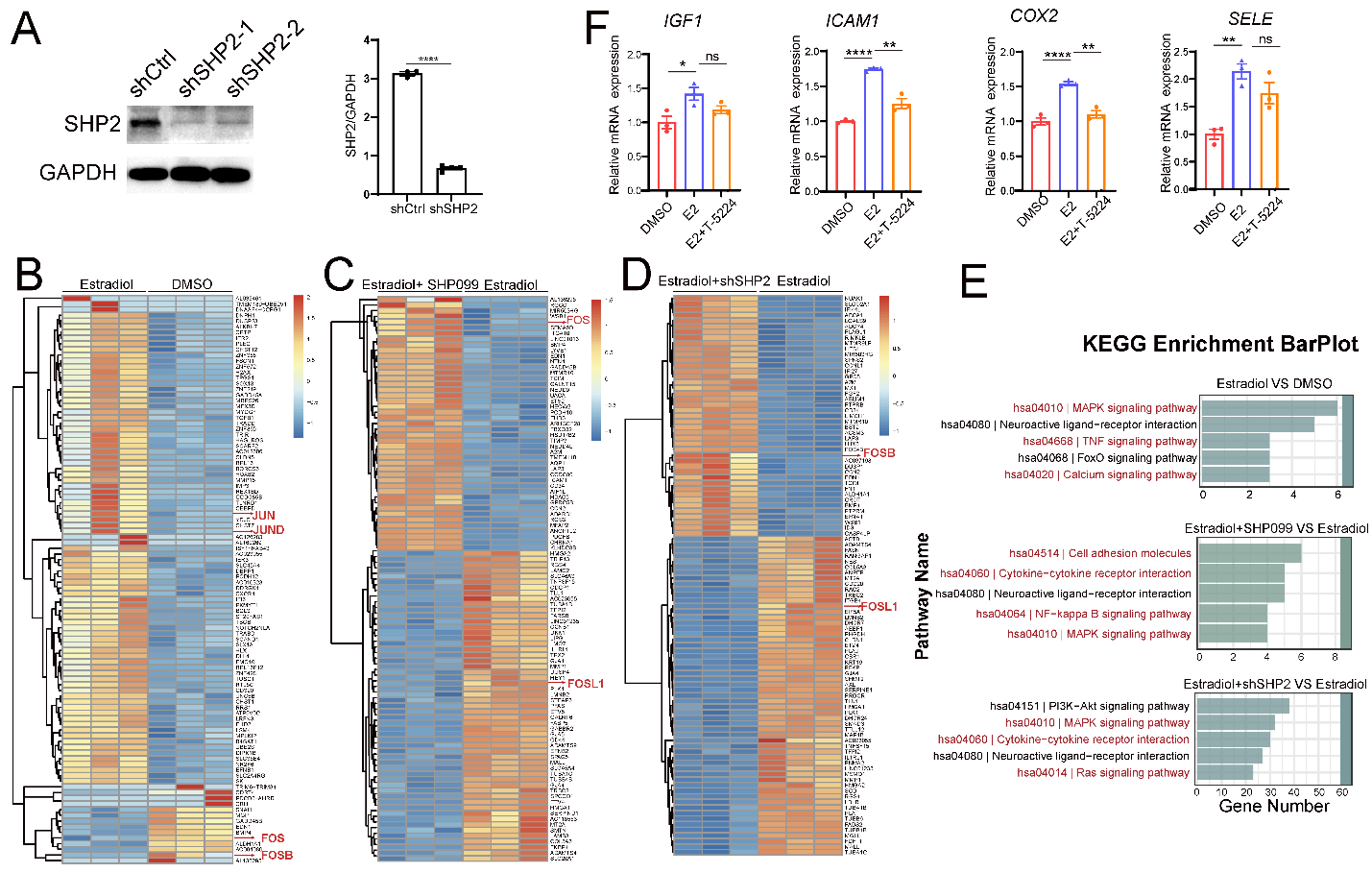


Figure S8. AP-1 transcription factor complex were involved in the regulation of SHP2 on endothelial cells.

**A** WB analysis of the SHP2 knockdown after shSHP2 virus infection in HUVECs. The protein expression was quantified. shCtrl, n=3; shSHP2, n=4. Student’s *t* test.

**B** Heatmap of top 100 differentially expressed genes in HUVECs between DMSO (dimethylsulfoxide) and estradiol (40 ng/mL) treated group. n=3/group.

**C** Heatmap of top 100 differentially-expressed genes in HUVECs between estradiol (40 ng/mL) and estradiol (40 ng/mL) plus SHP099 (5 μM) treated group. n=3/group.

**D** Heatmap of top 100 differentially-expressed genes after treated with estradiol (40 ng/mL) between WT HUVECs and SHP2-konckdown HUVECs. The HUVECs of the two groups were WT or SHP2 konckdown by virus infection (shSHP2). n=3/group.

**E** RNA-Seq analysis of KEGG pathways classifying differentially-expressed genes between E2 (40 ng/mL) and DMSO treated group (upper); estradiol (40 ng/mL) and estradiol (40 ng/mL) plus SHP099 (5 μM) treated group from RNA-Seq analysis (middle); estradiol (40 ng/mL) treated HUVECs and estradiol (40 ng/ml) treated HUVECs with SHP2 knockdown (bottom).

**F** Downstream gene expressions of AP-1 transcript complex in HUVECs treated with DMSO or E2 or E2 plus T5224 (10 μM). T5224 is a kind of AP-1 inhibitor, which specifically inhibits the DNA binding activity of c-Fos/c-Jun. All data are shown as the mean ± SEM. Data were analyzed by Tukey test. **P* value < 0.05; ** *P* value <0.01; **** *P* value <0.0001; ns represents no significant difference.


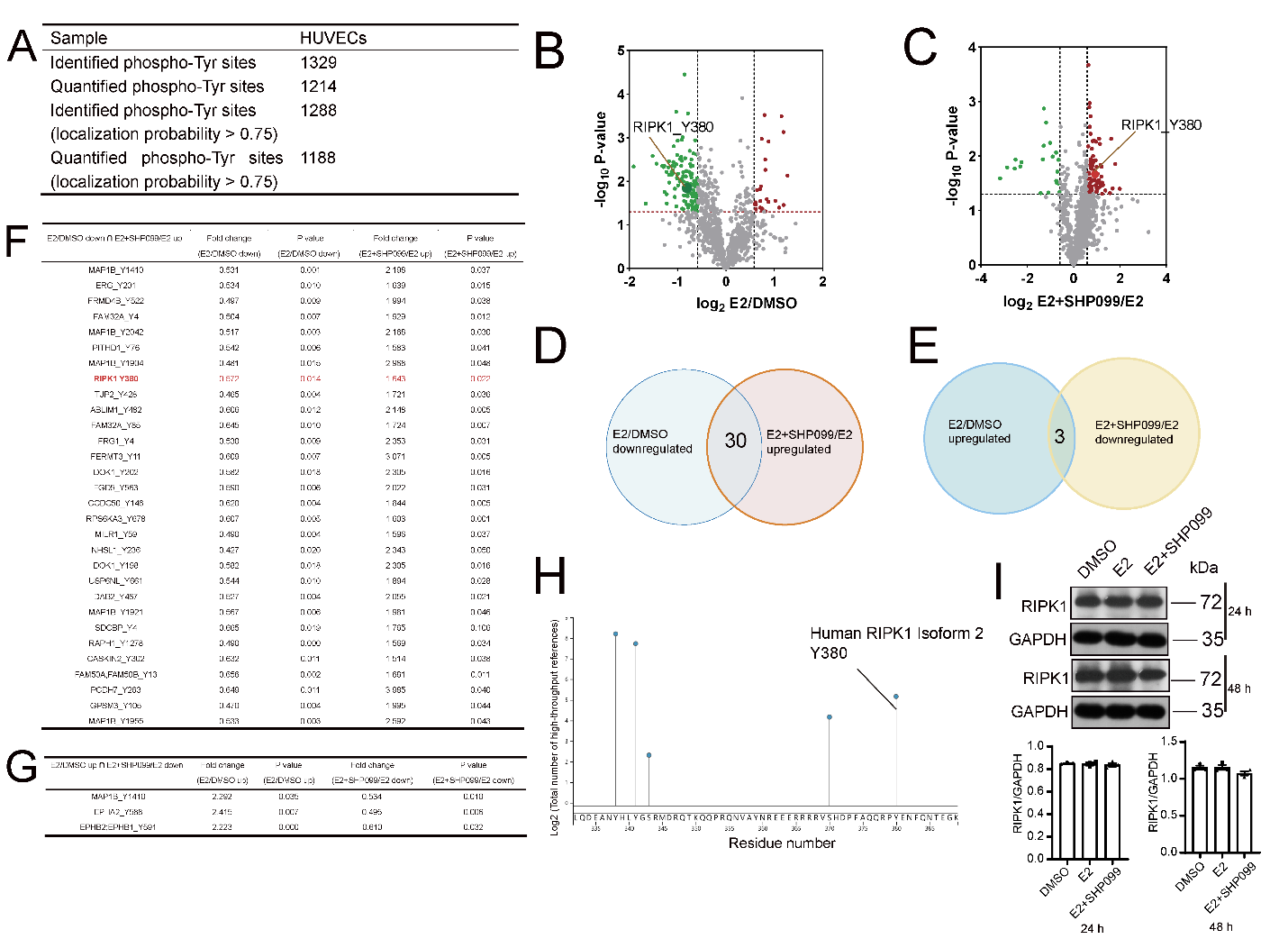


Figure S9. Detailed results of tyrosine phosphoproteomic in HUVECs

**A** The table showed the phosphorylated peptide and corresponding phosphorylation sites in HUVECs after indicated treatment detected by mass spectrometry.

**B** The volcano plot shows the upregulated (fold change > 1.5; *P* value <0.05) and downregulated (fold change < 0.6; *P* value <0.05) phosphorylation sites in HUVECs treated with estradiol (E2) or Vehicle control. n=3/group. Data were analyzed by Student’s *t* test.

**C** The volcano plot showed the upregulated (fold change > 1.5; *P* value <0.05) and downregulated (fold change < 0.6; *P* value <0.05) phosphorylation sites in HUVECs treated with E2 plus SHP099 or E2. E2 represents estradiol. n=3/group. Data were analyzed by Student’s *t* test.

**D** Venn diagram showed the phosphorylated site both in the set of downregulated sites of E2 compared DMSO and the upregulated sites of E2 plus SHP099 compared E2.

**E** Venn diagram showed the phosphorylated site both in the set of upregulated sites of E2 compared DMSO and the downregulated sites of E2 plus SHP099 compared E2.

**F** The table showed the detailed phosphorylated sites in the intersection of the Venn diagram in (D). The table showed the represent fold change value of 3 replicates. Student’s *t* test was used to calculate statistic difference.

**G** The table showed the detailed phosphorylated sites in the intersection of the Venn diagram in (E). The table showed the represent fold change value of 3 replicates. Student’s *t* test was used to calculate statistic difference.

**H** The phosphorylation sites of RIPK1 isoform2 from the PhosphoSitePlus database.

**I** The protein expression of total RIPK1 in HUVEC after indicated treatment for 24 h or 48 h. The protein expression of RIPK1 was quantified.


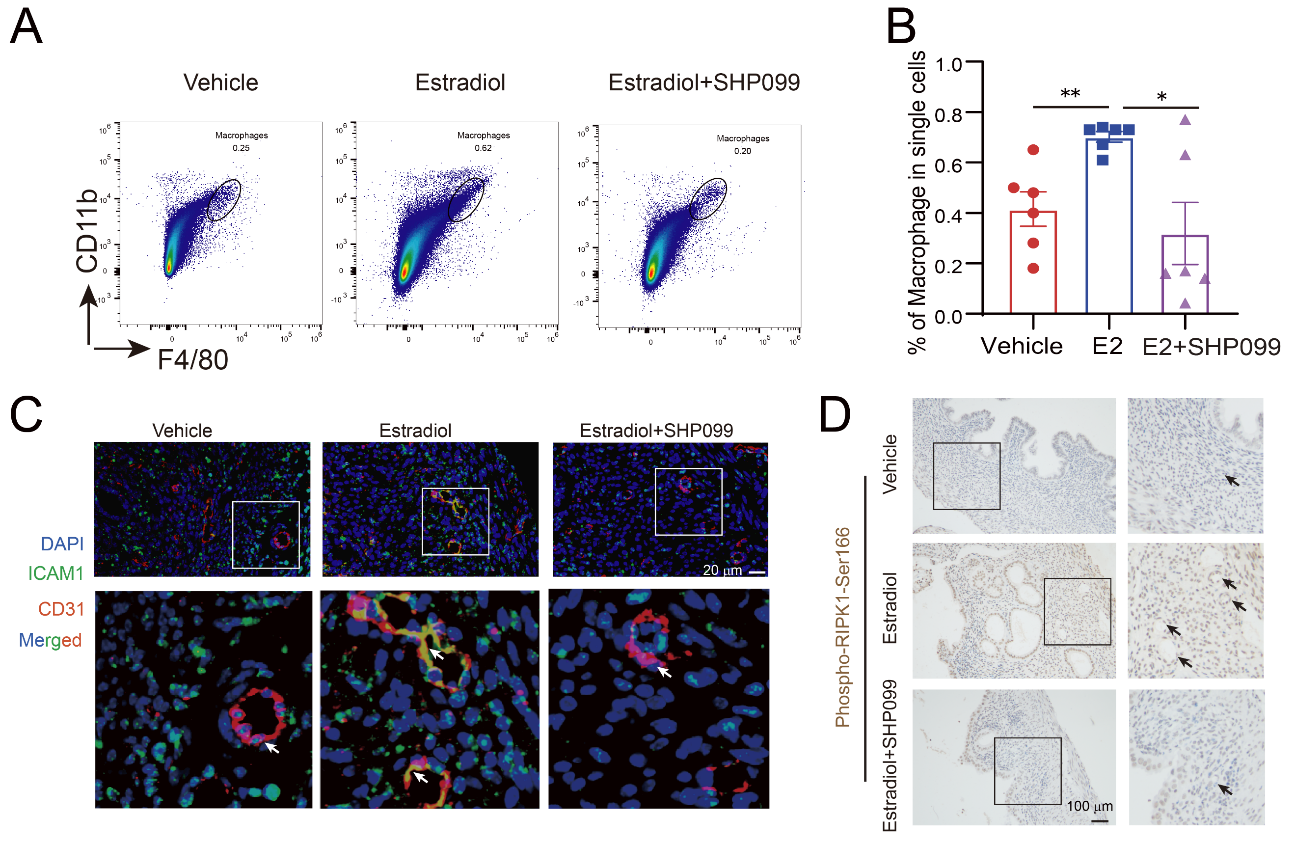


Figure S10. Validation of uterine macrophage, endothelial cell activation in EH mice model.

**A** Flow cytometry analysis of macrophages in the mice uteri after different treatment. Mice were treated with vehicle or estradiol or estradiol plus SHP099 for 21 days. Then collected the mice uteruses for flow cytometry.

**B** Statistical analysis of the percentage of tissue macrophages after indicated treatment in (A); n= 6 /group. E2 represents estradiol. Results are shown as the mean ± SEM. Data were analyzed by Tukey test. * *P* value < 0.05; ** *P* value <0.01.

**C** Representative immunofluorescence images of inflammatory activated endothelial cells (CD31^+^ICAM1^+^) in mice endometrium after indicated treatment. n=5/group.

**D** IHC staining of tissue protein levels of phosphor-RIPK1-Ser166 in mice uteruses treated with vehicle, estradiol or estradiol plus SHP099. n= 6 /group.

**Supplementary Table S1. Information for EH patients**

| Sample | Age | Clinical and Pathological diagnosis |
| --- | --- | --- |
| 1 | 53 | Broken endometrium with hyperplasia like changes |
| 2 | 54 | Endometrium with hyperplasia like changes, abnormal uterine bleeding |
| 3 | 74 | Endometrium hyperplasia, endometrial polyps |
| 4 | 33 | Endometrium hyperplasia, endometrial polyps |
| 5 | 48 | Focal squamous epithelial hyperplasia |
| 6 | 66 | Endometrium hyperplasia, endometrial polyps |
| 7 | 38 | Endometrial polyps, secretory phase like endometrium |
| 8 | 57 | Endometrium hyperplasia, endometrial polyps |
| 9 | 51 | Endometrium hyperplasia, endometrial polyps |
| 10 | 29 | Endometrium with hyperplasia like changes, endometrial polyps |
| 11 | 43 | Endometrium with hyperplasia like changes, endometrial polyps |
| 12 | 49 | Endometrium with secretory phase like changes, with local polypoid hyperplasia |
| 13 | 31 | Endometrium with hyperplasia like changes, uterus leiomyom |
| 14 | 50 | Endometrial polyps |
| 15 | 53 | Endometrium with secretory phase like changes, with local polypoid hyperplasia |
| 16 | 43 | Endometrium hyperplasia with focal polypoid hyperplasia |
| 17 | 56 | Endometrium hyperplasia, endometrial polyps |
| 18 | 36 | Complex hyperplasia, atypical hyperplasia in small focal areas |
| 19 | 47 | Simple endometrium hyperplasia, focal polypoid hyperplasia |
| 20 | 38 | Endometrium with hyperplasia like changes, endometrial polyps |

Supplementary Table S2. Antibodies

| Antibody | Cat number | | Dilution | | Source | | Company | |  |
| --- | --- | --- | --- | --- | --- | --- | --- | --- | --- |
| Anti-CD31 | Ab134168 | | WB 1:1000  IHC/IF 1:200 | | Rabbit | | Abcam | |  |
| Anti-CD31 | sc-18916 | | WB 1:1000  IHC/IF 1:100 | | Rat | | Santa Cruz | |  |
| Anti-SHP2 | sc-7384 | | WB 1:1000  IF 1:100 | | Mouse | | Santa Cruz | |  |
| Anti-pSHP2 Y542 | 3751S | | WB 1：1000 | | Rabbit | | Cell Signaling Technology | |  |
| Anti-pSHP2 Y580 | 5431T | | WB 1:1000 | | Rabbit | | Cell Signaling Technology | |  |
| Anti-ICAM1 | sc-8439 | | WB 1:1000  IF 1:100 | | Mouse | | Santa Cruz | |  |
| Anti-GAPDH | 60004-1-Ig | | WB 1:5000 | | Mouse | | Proteintech | |  |
| Anti-Tubulin | 66031-1-Ig | | WB 1:5000 | | Mouse | | Proteintech | |  |
| Anti-β-Actin | 66009-1-Ig | | WB 1:5000 | | Mouse | | Proteintech | |  |
| Anti-Ki67 | Ab15580 | | IHC 1:200 | | Rabbit | | Abcam | |  |
| Anti-IGF1 | A0830 | | WB 1:1000  IHC 1:150 | | Rabbit | | ABclonal | |  |
| Anti-VCAM1 | A0279 | | WB 1:1000 | | Rabbit | | ABclonal | |  |
| Anti-COX2 | 122827 | | IHC 1:200  WB 1:1000 | | Rabbit | | Cell Signaling Technology | |  |
| Anti-F4/80 | 30325T | | IHC 1:100 | | Rabbit | | Cell Signaling Technology | |  |
| Anti-CD68 | 76437S | | IF 1:250 | | Rabbit | | Cell Signaling Technology | |  |
| Anti-Lamin B | 13435 | | WB 1:1000 | | Rabbit | | Cell Signaling Technology | |  |
| Anti-p-c-FOS | 5348T | | WB 1:1000 | | Rabbit | | Cell Signaling Technology | |  |
| Anti-c-FOS | 2250T | | WB 1:1000 | | Rabbit | | Cell Signaling Technology | |  |
| Anti-p-c-JUN | 3270T | | WB 1:1000 | | Rabbit | | Cell Signaling Technology | |  |
| Anti-c-JUN | 9165T | | WB 1:1000 | | Rabbit | | Cell Signaling Technology | |  |
| Anti-RIPK1 | 3493T | | WB 1:1000 | | Rabbit | | Cell Signaling Technology | |  |
| Anti-ERα | | 13258S | | IF 1:200 | | Rabbit | | Cell Signaling Technology | |
| Anti-EpCAM | | 10483-1-AP | | IF 1:200 | | Mouse | | Proteintech | |
| Anti-phospho-RIPK1-Ser166 | AP1448 | | WB 1:1000  IHC 1:100 | | Rabbit | | ABclonal | |  |
| Anti-phospho-Tyrosine | AP0905 | | WB 1:1000  IP 1:50 | | Rabbit | | ABclonal | |  |
| Anti-Flag | 66008-4-Ig | | WB 1:1000  IP: 1:100 | | Mouse | | Proteintech | |  |
| Anti-HA | 3724S | | WB 1:1000  IP: 1:100 | | Mouse | | Cell Signaling Technology | |  |
| Anti-eGFP | 66002-1-Ig | | WB 1:1000 | | Mouse | | Proteintech | |  |
| Anti-ERK | sc-271269 | | WB 1:1000 | | Mouse | | Santa Cruz | |  |
| Anti-p-ERK | sc-7383 | | WB 1:1000 | | sc-7383 | | Santa Cruz | |  |
| Anti-Mouse IgG, Alexa Fluor 488 | A11029 | | IF 1:500 | | Goat | | Invitrogen | |  |
| Anti-Rabbit IgG, Alexa Fluor 488 | A11034 | | IF 1:500 | | Goat | | Invitrogen | |  |
| Anti-Mouse IgG, Alexa Fluor 594 | A21135 | | IF 1:500 | | Goat | | Invitrogen | |  |
| Anti-Rabbit IgG, Alexa Fluor 594 | A11012 | | IF 1:500 | | Goat | | Invitrogen | |  |
| Anti-mCD45 ef450 | 48-0451-82 | | 1:50 | | Rat | | Invitrogen | |  |
| Anti-mCD3 FITC | 100203 | | 1:50 | | Rat | | Biolegend | |  |
| Anti-CD11b PerCP-Cy5.5 | 45-0112-82 | | 1:50 | | Rat | | Invitrogen | |  |
| Anti-F4/80 APC | 17-4801-82 | | 1:50 | | Rat | | Invitrogen | |  |
| Anti-mCD31 PE | 160203 | | 1:50 | | Rabbit | | Biolegend | |  |

**Supplementary Table S3. PCR primers**

| Gene | Primer sequence 5'-3' |
| --- | --- |
| Human *PTPN11* | Forward: GAACTGTGCAGATCCTACCTCT |
|  | Reverse: TCTGGCTCTCTCGTACAAGAAA |
| Human *DUSP6* | Forward: GAAATGGCGATCAGCAAGACG |
|  | Reverse: CGACGACTCGTATAGCTCCTG |
| Human *DUSP11* | Forward: ATAGGTGCCGGGGACATTG |
|  | Reverse: TGGTTGCATGAGATGTGCTGA |
| Human *DUSP23* | Forward: GCCATTGCTGAAATCCGACG |
|  | Reverse: CTGCTCATAGGTCTCGATGGA |
| Human *CXCL10* | Forward: GTGGCATTCAAGGAGTACCTC |
|  | Reverse: TGATGGCCTTCGATTCTGGATT |
| Human *CXCL13* | Forward: GCTTGAGGTGTAGATGTGTCC |
|  | Reverse: CCCACGGGGCAAGATTTGAA |
| Human *IGF1* | Forward: GCTCTTCAGTTCGTGTGTGGA |
|  | Reverse: GCCTCCTTAGATCACAGCTCC |
| Human *PTX3* | Forward: TTATTCCCAATGCGTTCCAAGA |
|  | Reverse: GCACTAAAAGACTCAAGCCTCAT |
| Human OSM | Forward: CACAGACTGGCCGACTTAGAG |
|  | Reverse: AGTCCTCGATGTTCAGCCCA |
| Human WNT5A | Forward: GCCAGTATCAATTCCGACATCG |
|  | Reverse: TCACCGCGTATGTGAAGGC |
| Human FRZB | Forward: GAGCCCATACTCATCAAGTACCG |
|  | Reverse: CCTCGGGAGAGATGCACAC |
| Human *ICAM1* | Forward: TTGGGCATAGAGACCCCGTT |
|  | Reverse: GCACATTGCTCAGTTCATACACC |
| Human *SELE* | Forward: AGAGTGGAGCCTGGTCTTACA |
|  | Reverse: CCTTTGCTGACAATAAGCACTGG |
| Human *IL-6* | Forward: CCTGAACCTTCCAAAGATGGC |
|  | Reverse: TTCACCAGGCAAGTCTCCTCA |
| Human *IL-33* | Forward: GTGACGGTGTTGATGGTAAGAT |
|  | Reverse: AGCTCCACAGAGTGTTCCTTG |
| Human *NOS2* | Forward: TTCAGTATCACAACCTCAGCAAG |
|  | Reverse: TGGACCTGCAAGTTAAAATCCC |
| Human *IL1B* | Forward: ATGATGGCTTATTACAGTGGCAA |
|  | Reverse: GTCGGAGATTCGTAGCTGGA |
| Human *TNF* | Forward: GAGGCCAAGCCCTGGTATG |
|  | Reverse: CGGGCCGATTGATCTCAGC |
| Human *FOS* | Forward: CGCAGACTACGAGGCGTCATCC |
|  | Reverse: ATGGCAGTGACCGTGGGAATGA |
| Human *FOSB* | Forward: GCGATCACAACCAGCCAGGACC |
|  | Reverse: CCGCCACTGCTGTAGCCACTCAT |
| Human *FOSL1* | Forward: CACCCTCCCTAACTCCTTTCACCC |
|  | Reverse: GTCTCCGCTGCTGCTGCTACTCTT |
| Human PTGS2 | Forward: CAACAGAGTATGCGATGTGCTT |
|  | Reverse: CCTATCAGTATTAGCCTGCTTGTC |
| Mouse *Il1b* | Forward: GCAACTGTTCCTGAACTCAACT |
|  | Reverse: ATCTTTTGGGGTCCGTCAACT |
| Mouse *Tnfa* | Forward: CCCTCACACTCAGATCATCTTCT |
|  | Reverse: GCTACGACGTGGGCTACAG |
| Mouse *Il6* | Forward: TAGTCCTTCCTACCCCAATTTCC |
|  | Reverse: TTGGTCCTTAGCCACTCCTTC |
| *Shp2* for genotype identification | Forward: ACGTCATGATCCGCTGTCAG |
|  | Reverse: ATGGGAGGGACAGTGCAGTG |
| *Cdh5-ERT2Cre* for genotype identification | Forward: CCAAAATTTGCCTGCATTACCGGTCGATGC |
|  | Reverse: ATCCAGGTTACGGATATAGT |

**Supplementary Table S4. Endometrial organoid medium**

| **Product** | **Final Concentration** | **Supplier** |
| --- | --- | --- |
| DMEM/F-12，HEPES | 1X | Thermofisher |
| N2 supplement 100X | 1X | Gibco |
| B27 supplement minus vitamin A 50X | 1X | Gibco |
| N-Acetyl-L-cysteine | 1.25 mM | MCE |
| Insulin Transferrin Selenium 100X | 1X | Gibco |
| Wnt3a, Human | 200 ng/mL | MCE |
| Recombinant human EGF | 50 ng/mL | MCE |
| Recombinant human Noggin | 100 ng/mL | Peprotech |
| Recombinant human Rspondin-1 | 200 ng/mL | Peprotech |
| Recombinant Human FGF-basic (154 a.a.) | 2 ng/mL | Peprotech |
| Recombinant human FGF-10 | 100 ng/mL | MCE |
| Recombinant human HGF | 50 ng/mL | Peprotech |
| ALK-4, -5, -7 inhibitor, A83-01 | 500 nM | MCE |
| Nicotinamide | 2 mM | MCE |
| Y-27632 （ROCK inhibitor） | 10 mM | MCE |
| SB202190 (p38i) | 10 mM | MCE |
| GlutaMax | 2 mM | Gibco |
| 17-β Estradiol | 1 nM | MCE |
