# Supplementary material for: SHP2‐Triggered Endothelial Cell Activation Fuels Estradiol‐Independent Endometrial Sterile Inflammation
Source: Adv Sci (Weinh). 2024 Sep 5;11(41):2403038. doi: 10.1002/advs.202403038 (PMC11538683; doi:10.1002/advs.202403038)
Supplement: Supplementary file 2 — Supporting Information [file ADVS-11-2403038-s003.pdf]

ADVANCED SCIENCE
Open Access

Supporting Information

for *Adv. Sci.*, DOI 10.1002/advs.202403038

SHP2-Triggered Endothelial Cell Activation Fuels Estradiol-Independent Endometrial Sterile Inflammation

Jie Pan, Jiao Qu, Wen Fang, Lixin Zhao, Wei Zheng, Linhui Zhai, Minjia Tan, Qiang Xu, Qianming Du, Wen Lv* and Yang Sun**

Cell Line Authentication Service
STR Profile Report

**Sample Submitted By:**	Dr. Yang Sun<br>Nanjing University
**Email Address:**	yangsun@nju.edu.cn
**Sales Order:**	240720B 240720D
**Cell Line Designation:**	Endometrial organoid-1 Primary endometrial tissue-1
**Date Sample Received:**	Jul 20th, 2024 Jul 20th, 2024
**Report Date:**	Jul 22th, 2024

Methodology: Twenty-one short tandem repeat (STR) loci plus the Amelogenin locus were amplified using the commercially available SiFaSTR™ 23 plex Kit. The cell line sample was processed using the ABI Prism® 3130 XL Genetic Analyzer. Data were analyzed using GeneMapper® ID v3.2 software (Applied Biosystems). Appropriate positive and negative controls were run and confirmed for each sample submitted.

Data Interpretation: Cell lines were authenticated using Short Tandem Repeat (STR) analysis as described in 2021 in ANSI Standard (ASN-0002) by the ATCC Standards Development Organization (SDO) and in Jamie L. Almeida et al., Authentication of Human and Mouse Cell Lines by Short Tandem Repeat (STR) DNA Genotype Analysis. Assay Guidance Manual. PMID: 23805434. Bookshelf ID: NBK144066.

GTB™ performs STR Profiling following ISO 9001:2008 and ISO/IEC 17025:2005 quality standards. There are no warranties with respect to the services or results supplied, express or implied, including, without limitation, any implied warranty of merchantability or fitness for a particular purpose. Genetic Testing Biotechnology (GTB) is not liable for any damages or injuries resulting from receipt and/or improper, inappropriate, negligent or other wrongful use of the test results supplied, and/or from misidentification, misrepresentation, or lack of accuracy of those results. Your exclusive remedy against GTB and those supplying materials used in the services for any losses or damage of any kind whatsoever, whether in contract, tort, or otherwise, shall be, at GTB's option, refund of the fee paid for such service or repeat of the service.

NOTE: According to the recommendations of *IJC* on cell line authentication, the report is valid for 3 years since the issue date.

Technical Questions?
GTB Technical Support
+86-512-67486171
service@jsdna.org
Section 505, Yixin BLD
SIP, Suzhou, 215123
Jiangsu, P.R. China

Ordering Questions?
order@jsdna.org
GTB Corporation
+86-512-62806339
Section 303, Yixin BLD
SIP, Suzhou, 215123
Jiangsu, P.R. China

GENETIC TESTING BIOTECHNOLOGY

Cell Line Authentication Service
STR Profile Report

Sales Order: 240720B 240720D

Loci	Endometrial organoid-1		Primary endometrial tissue-1		Matching Probability
Amelogenin	X		X		/
D3S1358	16	17	16	17	0.1188
D5S818	9	11	9	11	0.0363
D2S1338	18	22	18	22	0.0202
TPOX	11		11		0.0400
CSF1PO	10		10		0.0420
Penta D	10	11	10	11	0.0396
TH01	9		9		0.2550
vWA	14	19	14	19	0.0800
D7S820	9	13	9	13	0.0032
D21S11	30	33.2	30	33.2	0.0372
Penta E	12	16	12	16	0.0168
D10S1248	12	13	12	13	0.0594
D8S1179	13	14	13	14	0.0988
D1S1656	15	16	15	16	0.0007
D18S51	15	19	15	19	0.0074
D12S391	19	20	19	20	0.0883
D6S1043	12	19	12	19	0.0425
D19S433	13	16	13	16	0.0061
D16S539	12	13	12	13	0.0608
D13S317	10		10		0.0169
FGA	22	26	22	26	0.0090
LR					6.7903×10^{32}

Explanation of Test Results

- [x] The two submitted sample profiles are human without any other human contamination, but not a match for any profile in the ExPASy STR database.
- [x] The DNA typing results support that the two submitted samples are from same individual with a LR= 6.7903×10^{32}
- [ ] The DNA typing results support that the submitted samples are from different individuals.

e-Signature, Technician:

Digitally signed by Xuekun Chen
DN: cn=Xuekun Chen, o=Genetic Testing Biotechnology (Suzhou), ou=DNA Typing Section, email=order@jsdna.org, c=CN
Date: 2024.07.22 15:41:44 +08'00'

e-Signature, Reviewer:

Digitally signed by Xiankun Zhao
DN: cn=Xiankun Zhao, o=Genetic Testing Biotechnology (Suzhou), ou=Supervision Section, email=service@jsdna.org, c=CN
Date: 2024.07.22 15:44:36 +08'00'

GENETIC TESTING BIOTECHNOLOGY — GUARANTEE — CELL LINE AUTHENTICATION — SINCE 2013 SUZHOU

More information

Addendum: Electropherogram for the customer's sample set 1 of 1

Cell Line Authentication Service
STR Profile Report

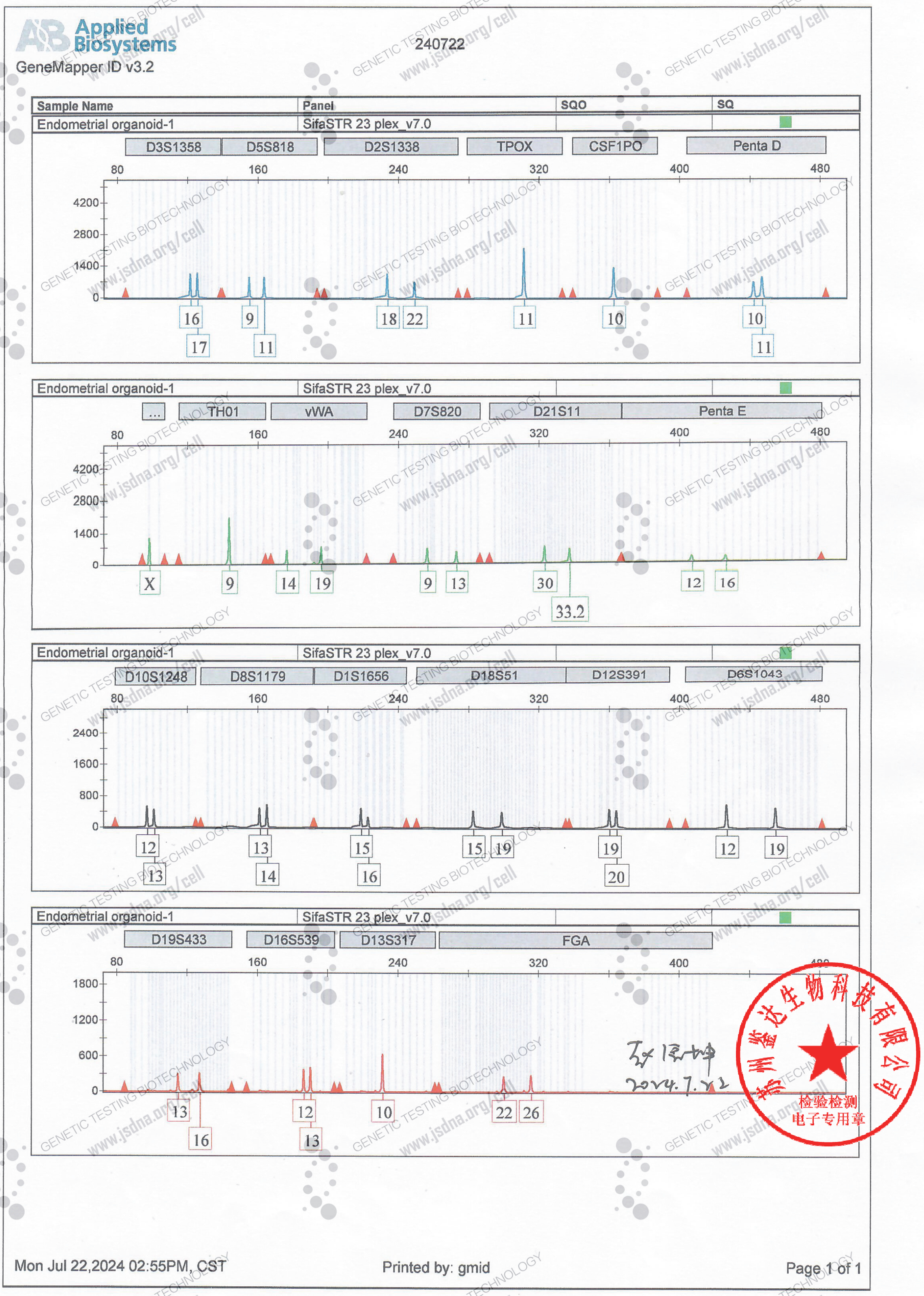

GENETIC TESTING BIOTECHNOLOGY www.jsdna.org/cell

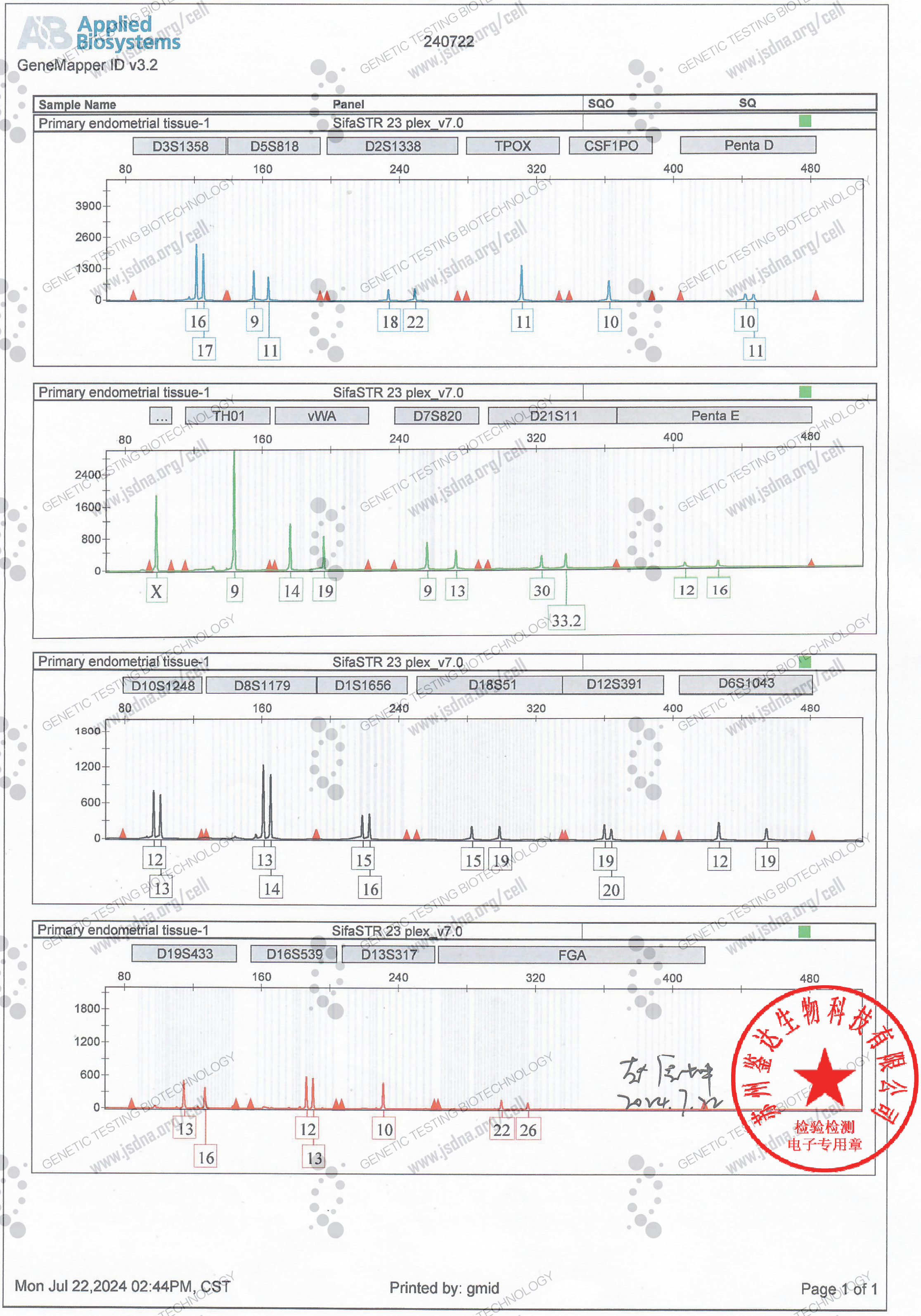
Applied Biosystems
GeneMapper ID v3.2
240722
Sample Name
Panel
SQO
SQ
Primary endometrial tissue-1
SifaSTR 23 plex_v7.0
D3S1358
D5S818
D2S1338
TPOX
CSF1PO
Penta D
16
17
9
11
18
22
11
10
10
11
TH01
vWA
D7S820
D21S11
Penta E
X
9
14
19
9
13
30
33.2
12
16
D10S1248
D8S1179
D1S1656
D18S51
D12S391
D6S1043
12
13
13
14
15
16
15
19
19
20
12
19
D19S433
D16S539
D13S317
FGA
13
16
12
13
10
22
26
Mon Jul 22,2024 02:44PM, CST
Printed by: gmid
Page 1 of 1
