# Supplementary material for: SHP2‐Triggered Endothelial Cell Activation Fuels Estradiol‐Independent Endometrial Sterile Inflammation
Source: Adv Sci (Weinh). 2024 Sep 5;11(41):2403038. doi: 10.1002/advs.202403038 (PMC11538683; doi:10.1002/advs.202403038)

ADVANCED SCIENCE
Open Access

Supporting Information

for *Adv. Sci.*, DOI 10.1002/advs.202403038

SHP2-Triggered Endothelial Cell Activation Fuels Estradiol-Independent Endometrial Sterile Inflammation

Jie Pan, Jiao Qu, Wen Fang, Lixin Zhao, Wei Zheng, Linhui Zhai, Minjia Tan, Qiang Xu, Qianming Du, Wen Lv* and Yang Sun**

<u>Cell Line Authentication Service</u>
STR Profile Report

GENETIC TESTING BIOTECHNOLOGY

**Sample Submitted By:**	Dr. Yang Sun<br>Nanjing University
**Email Address:**	yangsun@nju.edu.cn
**Sales Order:**	240720C 240720E
**Cell Line Designation:**	Endometrial organoid-2 Primary endometrial tissue-2
**Date Sample Received:**	Jul 20th, 2024 Jul 20th, 2024
**Report Date:**	Jul 22th, 2024

Methodology: Twenty-one short tandem repeat (STR) loci plus the Amelogenin locus were amplified using the commercially available SiFaSTR™ 23 plex Kit. The cell line sample was processed using the ABI Prism® 3130 XL Genetic Analyzer. Data were analyzed using GeneMapper® ID v3.2 software (Applied Biosystems). Appropriate positive and negative controls were run and confirmed for each sample submitted.

Data Interpretation: Cell lines were authenticated using Short Tandem Repeat (STR) analysis as described in 2021 in ANSI Standard (ASN-0002) by the ATCC Standards Development Organization (SDO) and in Jamie L. Almeida et al., Authentication of Human and Mouse Cell Lines by Short Tandem Repeat (STR) DNA Genotype Analysis. Assay Guidance Manual. PMID: 23805434. Bookshelf ID: NBK144066.

GTB™ performs STR Profiling following ISO 9001:2008 and ISO/IEC 17025:2005 quality standards.
There are no warranties with respect to the services or results supplied, express or implied, including, without limitation, any implied warranty of merchantability or fitness for a particular purpose. Genetic Testing Biotechnology (GTB) is not liable for any damages or injuries resulting from receipt and/or improper, inappropriate, negligent or other wrongful use of the test results supplied, and/or from misidentification, misrepresentation, or lack of accuracy of those results. Your exclusive remedy against GTB and those supplying materials used in the services for any losses or damage of any kind whatsoever, whether in contract, tort, or otherwise, shall be, at GTB's option, refund of the fee paid for such service or repeat of the service.

NOTE: According to the recommendations of *IJC* on cell line authentication, the report is valid for 3 years since the issue date.

Technical Questions?
GTB Technical Support
+86-512-67486171
service@jsdna.org
Section 505, Yixin BLD
SIP, Suzhou, 215123
Jiangsu, P.R. China

Ordering Questions?
order@jsdna.org
GTB Corporation
+86-512-62806339
Section 303, Yixin BLD
SIP, Suzhou, 215123
Jiangsu, P.R. China

GENETIC TESTING BIOTECHNOLOGY

Cell Line Authentication Service
STR Profile Report

Sales Order: 240720C 240720E

Loci	Endometrial organoid-2		Primary endometrial tissue-2		Matching Probability
Amelogenin	X		X		/
D3S1358	16		16		0.0729
D5S818	12		12		0.0529
D2S1338	18	24	18	24	0.0295
TPOX	9	11	9	11	0.0600
CSF1PO	11	12	11	12	0.1518
Penta D	13	14	13	14	0.0108
TH01	6	9	6	9	0.0960
vWA	14	18	14	18	0.0675
D7S820	11	12	11	12	0.1538
D21S11	30		30		0.0961
Penta E	11		11		0.0328
D10S1248	14		14		0.0595
D8S1179	13	14	13	14	0.0988
D1S1656	16	17	16	17	0.0377
D18S51	12	19	12	19	0.0002
D12S391	22	24	22	24	0.0016
D6S1043	12	18	12	18	0.0483
D19S433	12	15	12	15	0.0064
D16S539	9	11	9	11	0.1397
D13S317	9		9		0.0169
FGA	21	23	21	23	0.0552
LR					1.0082×10^{31}

Explanation of Test Results

- [x] The two submitted sample profiles are human without any other human contamination, but not a match for any profile in the ExPASy STR database.
- [x] The DNA typing results support that the two submitted samples are from same individual with a LR= 1.0082×10^{31}
- [ ] The DNA typing results support that the submitted samples are from different individuals.

e-Signature Technician: Digitally signed by Xuekun Chen
DN: cn=Xuekun Chen, o=Genetic Testing Biotechnology (Suzhou), ou=DNA Typing Section, email=order@jsdna.org, c=CN
Date: 2024.07.22 15:43:54 +08'00'

e-Signature Reviewer: Digitally signed by Xiankun Zhao
DN: cn=Xiankun Zhao, o=Genetic Testing Biotechnology (Suzhou), ou=Supervision Section, email=service@jsdna.org, c=CN
Date: 2024.07.22 15:44:19 +08'00'

GENETIC TESTING BIOTECHNOLOGY — GUARANTEE — CELL LINE AUTHENTICATION

More information

Addendum: Electropherogram for the customer's sample set 1 of 1

Cell Line Authentication Service
STR Profile Report

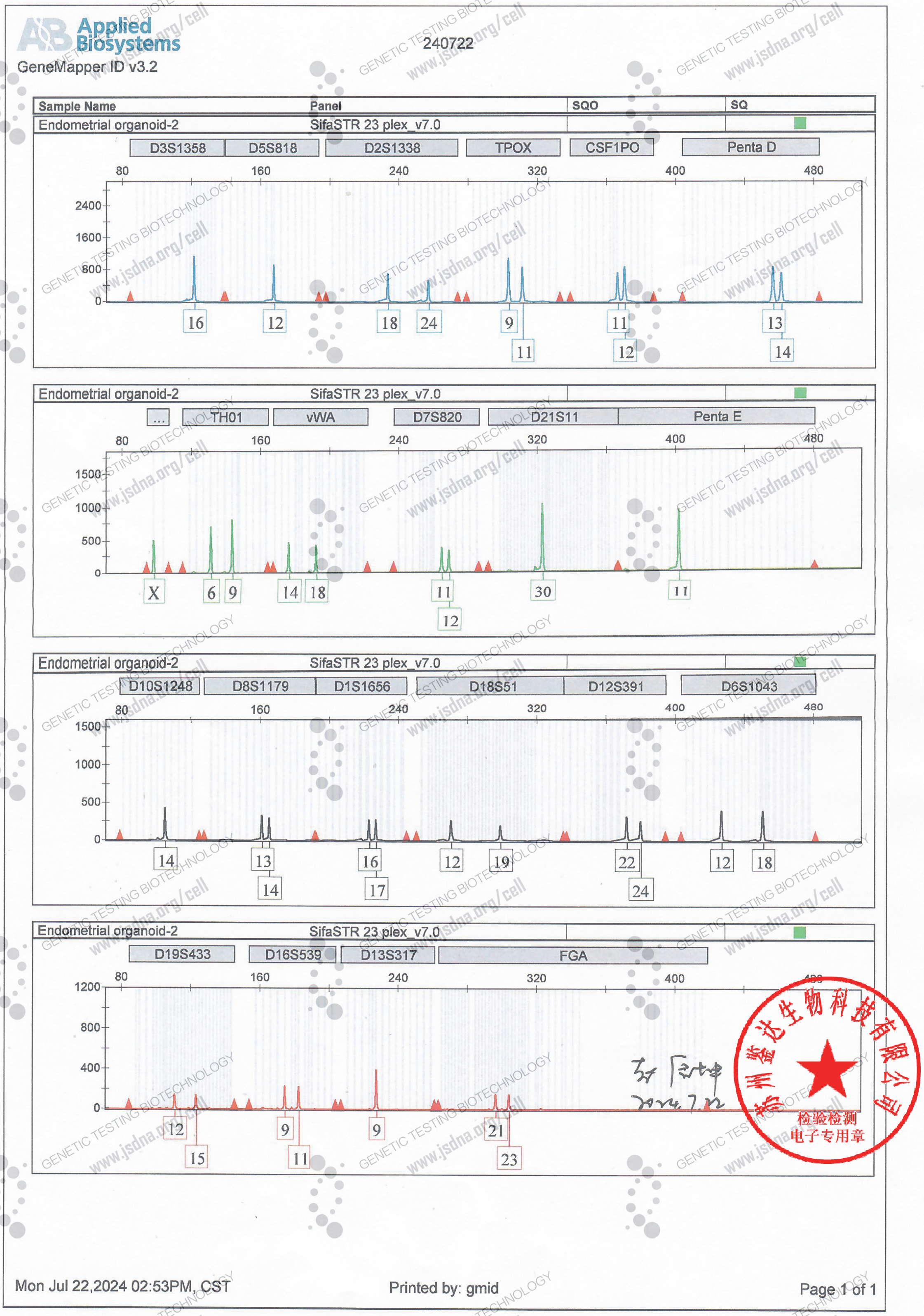

GENETIC TESTING BIOTECHNOLOGY

Cell Line Authentication Service
STR Profile Report

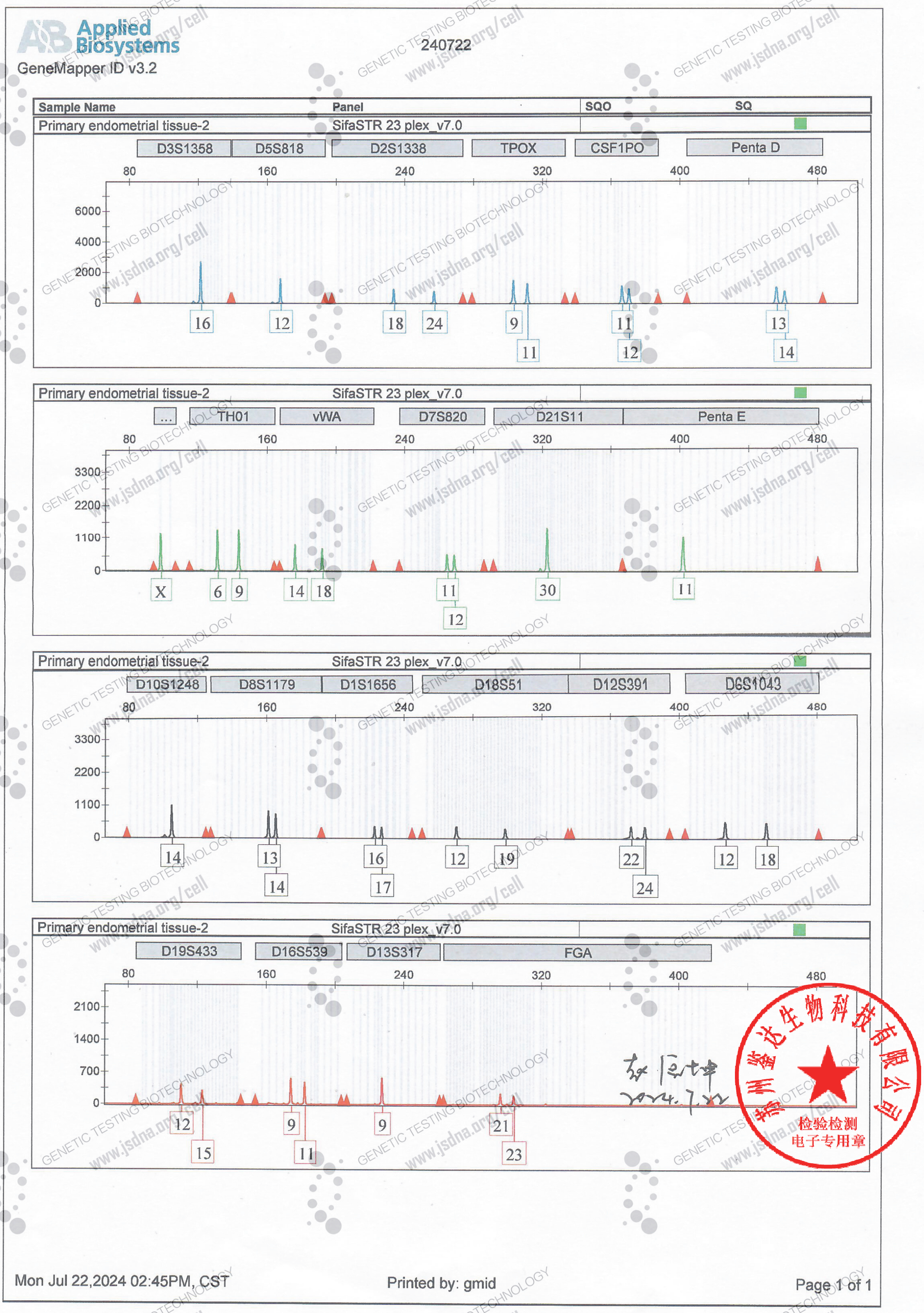

Supplement: Supplementary file 3 — Supporting Information [file ADVS-11-2403038-s004.pdf]
